# Enhancing Maturation and Translatability of Human Pluripotent Stem Cell-Derived Cardiomyocytes through a Novel Medium Containing Acetyl-CoA Carboxylase 2 Inhibitor

**DOI:** 10.3390/cells13161339

**Published:** 2024-08-13

**Authors:** Cláudia Correia, Jonas Christoffersson, Sandra Tejedor, Saïd El-Haou, Meztli Matadamas-Guzman, Syam Nair, Pierre Dönnes, Gentian Musa, Mattias Rohman, Monika Sundqvist, Rebecca B. Riddle, Bramasta Nugraha, Ioritz Sorzabal Bellido, Markus Johansson, Qing-Dong Wang, Alejandro Hidalgo, Karin Jennbacken, Jane Synnergren, Daniela Später

**Affiliations:** 1 Research and Early Development, Cardiovascular, Renal and Metabolism (CVRM), BioPharmaceuticals R&D, AstraZeneca, 43150 Gothenburg, Swedenalejandro.hidalgogon@mcri.edu.au (A.H.);; 2 Systems Biology Research Center, School of Bioscience, University of Skövde, 54128 Skövde, Sweden; 3 Mechanistic Biology and Profiling, Discovery Sciences, AstraZeneca R&D, Cambridge CB2 0AA, UK; 4 SciCross AB, 54135 Skövde, Sweden; 5 Discovery Sciences, BioPharmaceuticals R&D, AstraZeneca, 43150 Gothenburg, Sweden; 6 Department of Pharmacology, University of Cambridge, Cambridge CB2 1PD, UK; 7 Data Sciences and Quantitative Biology, Discovery Sciences, AstraZeneca R&D, Cambridge CB2 0AA, UK; 8 Integrated Cardio Metabolic Centre (ICMC), Department of Medicine, Karolinska Institute, Blickagången 6, 14157 Huddinge, Sweden; 9 Department of Molecular and Clinical Medicine, Institute of Medicine, Sahlgrenska Academy, University of Gothenburg, 41345 Gothenburg, Sweden

**Keywords:** human pluripotent stem cell-derived cardiomyocyte (hPSC-CM) maturation, acetyl-CoA carboxylase 2 (ACC2), cardiac hypertrophy, translatable in vitro model, in vitro-to-in vivo correlation

## Abstract

Human pluripotent stem cell-derived cardiomyocytes (hPSC-CMs) constitute an appealing tool for drug discovery, disease modeling, and cardiotoxicity screening. However, their physiological immaturity, resembling CMs in the late fetal stage, limits their utility. Herein, we have developed a novel, scalable cell culture medium designed to enhance the maturation of hPSC-CMs. This medium facilitates a metabolic shift towards fatty acid utilization and augments mitochondrial function by targeting Acetyl-CoA carboxylase 2 (ACC2) with a specific small molecule inhibitor. Our findings demonstrate that this maturation protocol significantly advances the metabolic, structural, molecular and functional maturity of hPSC-CMs at various stages of differentiation. Furthermore, it enables the creation of cardiac microtissues with superior structural integrity and contractile properties. Notably, hPSC-CMs cultured in this optimized maturation medium display increased accuracy in modeling a hypertrophic cardiac phenotype following acute endothelin-1 induction and show a strong correlation between in vitro and in vivo target engagement in drug screening efforts. This approach holds promise for improving the utility and translatability of hPSC-CMs in cardiac disease modeling and drug discovery.

## 1. Introduction

The advent of human induced pluripotent stem cells (hiPSCs) has revolutionized cardiovascular research, providing a versatile platform for generating cardiomyocyte-like cells from both healthy and patient-specific sources. This advancement offers unprecedented opportunities for studying cardiac development, modeling cardiac diseases, and paving the way for personalized therapies. Despite achieving high yields and purity, current differentiation protocols often result in human pluripotent stem cell-derived cardiomyocytes (hPSC-CMs) that exhibit fetal-like characteristics, limiting their utility in advancing cardiovascular therapies. Enhancing the maturation of hPSC-CMs is crucial for creating more physiologically relevant models for studying heart diseases and increasing translatability and predictability to humans in drug discovery, screening, and cardiotoxicity testing.

In the past few years, efforts have been made to shed light on the mechanisms driving hPSC-CM maturation. Various approaches have been investigated and successfully shown the capability to propel hPSC-CM maturation forward, including long-term culture [1,2], exposure to specific growth/signaling factors [3,4,5,6,7], three-dimensional (3D) culture [8,9,10,11], mechanical and electrical stimulation [12], genetic and microRNA interventions [13,14], and metabolic modulation through carbon source switches or specific factors like PPARdelta activators [15,16,17,18,19,20,21].

Despite these advancements, achieving a truly adult-like CM phenotype in vitro remains elusive, suggesting that a multifactorial approach may be necessary [22,23,24].

A critical aspect of CM maturation that has gained attention is the role of mitochondrial function. Mitochondrial network remodeling and oxidative metabolism are essential for achieving a mature phenotype, as evidenced by the shift in energy production from mainly anaerobic glycolysis and lactate oxidation in fetal CMs to 70–90% fatty acid oxidation (FAO) in adult CMs [25,26]. Activating mitochondrial function by exposing hPSC-CMs to a glucose-depleted, galactose- and FA-enriched medium has shown promise in inducing a metabolic shift towards a more mature hPSC-CM metabolism and function [15,27].

Herein, we have explored a novel approach to further enhance mitochondrial function and FAO through the inhibition of Acetyl-CoA carboxylase 2 (ACC2), a key enzyme involved in the regulation of FA metabolism and energy homeostasis [28]. ACC2, predominantly found in heart and muscle tissues, is associated with the mitochondrial outer membrane, and functions as a suppressor of mitochondrial FAO by inhibiting carnitine palmitoyl transferase 1 (CPT-1), essential for the transport of long-chain acyl-CoA into the mitochondrial membrane for β-oxidation. Inhibition of ACC2 reduces malonyl-CoA levels, thus relieving the inhibition of CPT-1 and enhancing the transport and oxidation of FAs within mitochondria. Previous studies have demonstrated the benefits of ACC2 deletion in rodent models with existing cardiac pathology, including increased cardiac FAO [29], improved cardiac energetics and function [30], and attenuation of cardiac hypertrophy [29,31].

Building on this foundation, we investigated for the first time a synergistic approach, integrating pharmacological inhibition of ACC2 with biochemical and metabolic stimuli, to foster and expedite hPSC-CM maturation.

We present a straightforward and robust maturation protocol that improves the metabolic, functional, electrophysiological, structural, and molecular maturation of 2D hPSC-CMs. This approach also facilitates the generation of cardiac 3D microtissues with superior structure and contractile kinetics versus cardiac microtissues cultured in standard glucose-based medium. HiPSC-CMs cultured in this enhanced maturation medium exhibited increased accuracy in modeling an early hypertrophic response upon endothelin-1 (ET-1) stimulation. Furthermore, mature hiPSC-CMs demonstrated a reliable in vitro-to-in vivo translatability of target engagement following branched-chain keto acid (BCKA) reduction upon branched-chain keto acid dehydrogenase kinase (BCKDK) inhibition, a feature not attainable with hiPSC-CMs cultured in standard medium.

## 2. Materials and Methods

### 2.1. Cell Lines and Reagents

CMs derived from three hiPS cell lines and one hES cell line were used in these experiments: iCell cardiomyocytes^2^ (product number R1017, Fujifilm Cellular Dynamics, Madison, WI, USA), ChiPS22 (product number Y00325, Takara Bio Europe, Swedish Filial, Gothenburg, Sweden), iPSC C32 (AIBN, The University of Queensland, Brisbane, Australia [32]) and SA121 (product number Y00020, Takara Bio Europe). 

Unless otherwise noted, all reagents were purchased from SigmaAldrich (Schnelldorf, Germany).

### 2.2. Cell Culture

#### 2.2.1. iCell

iCell cardiomyocytes^2^ (iCell CMs^2^, cryopreserved at day 30–32 of differentiation) were thawed, resuspended in Plating Medium (M1001, Fujifilm Cellular Dynamics), and seeded at 150,000 cells/cm^2^ in fibronectin-coated well plates. The Plating Medium was changed to maintenance medium (M1003, Fujifilm Cellular Dynamics) after 24 h and changed again to maturation medium after 72 h. 

#### 2.2.2. ChiPS22

CMs derived from ChiPS22 (cryopreserved at day 19 of differentiation) were thawed, resuspended in Thawing Medium (Cellartis CM Thawing Medium Y10062 TakaraBio Europe), and seeded at 150,000 cells/cm^2^ in fibronectin-coated wells. The Thawing Medium was changed to maintenance medium (Cellartis CM Culture Medium Y10063 TakaraBio Europe) after 24 h and changed again to maturation medium after 72 h. 

#### 2.2.3. iPSC C32

The C32 hiPSC line was maintained in E8 medium and differentiated as previously described [33]. Briefly, cells were plated in Matrigel-coated plates at a cell density of 5.0 × 10^5^–1.0 × 10^6^ per well in a 12-well plate two days before starting differentiation. On day 0, E8 medium was replaced with RPMI/B27 (minus insulin) containing 1 μM GSK3 inhibitor CHIR 98014 (Selleckchem, Cologne, Germany) for 24 h. On day 1, medium was replaced with RPMI/B27 (minus insulin). On day 3, half of the medium was changed to the RPMI/B27 medium containing 2 μM Wnt-C59 (Selleckchem), which was then completely removed during the medium change on day 5. On day 5, medium was changed to RPMI/B27 (plus insulin) and changed every other day until day 15 of differentiation. On day 15, medium was changed to maturation medium.

#### 2.2.4. hESC SA121

The SA121 hESC line was maintained in mTeSR^TM^1 culture medium (STEM CELL Technologies) and differentiated as previously described [34]. Briefly, cells were seeded in 12-well plates coated with Matrigel and maintained in mTeSR^TM^1 for 2 days. Then, CHIR99021 and Wnt-C59 small molecules (both from TOCRIS, Abingdon, UK) diluted in RPMI/B27 (minus insulin) medium were used to canonically activate and inhibit the Wnt signaling pathway, respectively. On day 8 of differentiation, medium was changed to RPMI/B27 (plus insulin) and changed every other day until day 15 of differentiation. On day 15, medium was changed to maturation medium.

### 2.3. Maturation Protocol

hPSC-CM maturation was initiated by changing the standard cell culture medium to maturation medium (RPMI no glucose supplemented with B27 (plus insulin), 100 µM oleic acid, 50 µM palmitic acid, 10 mM galactose, 1 µM dexamethasone, 100 nM T3, 100 ng/mL IGF1 (BioTechne, Dublin, Ireland), and 5 µM ACC2 inhibitor (CP640186). For experiments using the C32 hiPSC line, DMEM no glucose was used instead of RPMI. Culture medium was replaced every two to three days, and experiments were conducted after two weeks in maturation medium. 

### 2.4. In Vitro Hypertrophy Cell Model

After 2 weeks in either standard or maturation media, medium was switched to hypertrophy stimulation medium composed of RPMI no glucose supplemented with B27 plus insulin, 5.5 mM glucose and 10 nM endothelin-1 (ET-1, E7764). hPSC-CMs were cultured for 48 h in this medium before their use for further analyses.

### 2.5. Characterization of hPSC-CMs

#### Bioenergetics Seahorse Analysis

The Seahorse XF96 extracellular flux analyzer (Agilent, Santa Clara, CA, USA) was used to assess mitochondrial function. Seahorse plates were coated with fibronectin, and hiPSC-CMs were seeded onto the plates at a density of 30,000 cells per well. Culture medium was exchanged for Agilent Seahorse XF RPMI Basal Medium supplemented with 5.5 mM glucose, 2 mM glutamine, 150 µM sodium pyruvate, 100 µM Palmitate/BSA, and 5 mM Hepes, 1 h before the assay and for the duration of the measurement. For the Mito Stress test, mitochondria inhibitors were injected during the measurements as follows: Oligomycin (2 µM), 2.4-Dinitrophenol (DNP, 225 µM), rotenone, and antimycin A (both at 1.11 µM). The oxygen consumption rate (OCR) and extracellular acidification rate (ECAR) values were normalized to the number of nuclei per well quantified by Hoechst staining and fluorescence imaging. The baseline OCR was defined as the average values measured from time points 2 to 4 during the experiments subtracting the non-mitochondrial oxygen consumption rate determined as the minimum rate measurement after rotenone/antimycin A injection. Spare respiratory capacity was calculated as the difference between uncoupler DNP and baseline OCR values. ATP production was calculated by subtracting the minimum rate measurement after Oligomycin injection from the last rate measurement before Oligomycin injection. Proton leak was calculated by subtracting non-mitochondrial OCR from the minimum rate measurement after Oligomycin injection. Lastly, oxidation of exogenous FA was measured after injection of 50 µM Etomoxir. OCR due to FA oxidation was determined by subtracting the minimum rate measurement after Etomoxir injection from the last rate measurement before Oligomycin injection. All wells with a basal respiratory rate below 40 pmol/min were excluded from analysis.

### 2.6. Structural Analysis

#### 2.6.1. Immunofluorescence Staining

Cells were seeded in 96-well CellCarrier Ultra Phenoplates (PerkinElmer, Waltham, MA, USA) coated with fibronectin at a density of 150,000 cells/cm^2^. After washing three times with PBS, cells were fixed with 4% paraformaldehyde (PFA, ThermoFisher Scientific, Waltham, MA, USA) in PBS for 20 min at 4 °C, followed by three washes of PBS. A solution consisting of 5% FBS and 0.3% Triton X-100 in PBS was used to block and permeabilize the cells at RT for 1 h. For immunostaining, samples were incubated with primary antibodies overnight at 4 °C. Antibodies were diluted in antibody diluent, containing 1% FBS and 0.1% Triton X-100 at the optimized concentration: Connexin43 (1:400, ab117843, Abcam, Cambridge, UK), α-sarcomeric actinin (1:400, A7811, Sigma-Aldrich, Schnelldorf, Germany), Phospholamban (1:500, MA3-922, ThermoFisher), SERCA2 (1:500, ab150435, Abcam), cTNT (1:500, MS-295, ThermoFisher). Samples were then washed with antibody diluent solutions 3 times, and secondary antibodies diluted 1:500 in antibodies diluent solution were added and incubated at RT for 2 h. Next, a solution containing Hoecht33342 (1:2000, ThermoFisher, PN # 62249) and Cellbrite^TM^ Cytoplasmic membrane dye (30022, Biotium, Fremont, CA, USA) was added, and samples were incubated at RT for 10 min, followed by three PBS washes. Cells were stored at 4 °C before analysis. 

#### 2.6.2. Confocal Imaging and Image Analysis

Imaging was performed on a Yokogawa CV7000 microscope in scanning confocal mode using a dual Nipkow disk. First, 96-well CellCarrier Ultra Phenoplates (PerkinElmer) were mounted on a motorized stage and images were acquired in a row-wise “zigzag” fashion at RT. The system’s CellVoyager CV7000 software and 405/488/561/640 nm solid laser lines were used to acquire single Z-plane 16-bit TIFF images through a dry 40× objective lens using a cooled sCMOS camera with 2560 × 2160 pixels and a pixel size of 6.5 μm without pixel binning. Nine images in a 3 × 3 orientation were acquired of the center from each well. Image segmentation and feature extraction was performed with Columbus.

Images (either a single Z-plane or a maximum projection, as indicated above) were analyzed in Columbus (v.2.8.2 and 2.8.3, Perkin Elmer), and data were processed in Spotfire (v.7.9.2, TIBCO) and Prism (v.8, GraphPad). For all cell imaging experiments, cell nuclei and cytoplasm were identified, and parameters measured within the total cell area, or a region within the cell area (nucleus, cytoplasm, or concentric zones radiating from the nucleus to the plasma membrane).

#### 2.6.3. Cell Morphology Analysis

Cell area, circularity index, and aspect ratio were determined with FIJI (ImageJ v.2.14.0) software using standard analysis plugins. For each condition, at least 60 cells were analyzed.

#### 2.6.4. Sarcomere Characterization

Characterization of sarcomeres was based on immunofluorescence staining for α-sarcomeric actinin and performed using previously established methods based on the Fast Fourier Transform (FFT) for measuring sarcomere length [35] and sarcomere orientation [36]. The alignment of sarcomeres was quantified using the anisotropy index derived from the FFT spectrum. The anisotropy index measures the directional uniformity of the sarcomere network, ranging from 0, for randomly oriented sarcomeres, to 1, representing a perfectly aligned network. For each condition, at least 70 cells were analyzed from 3 separate experiments. SarcGraph, a tool for automatic detection, tracking, and analysis of Z-disks [37], was used to quantify Z-band width as a measurement of myofibrillar density. 

### 2.7. Ultrastructural Analysis

#### 2.7.1. TEM Analysis

iCell CMs^2^ and ChiPS22-derived CMs were seeded in 8-well chamber slides (Permanox, 177445PK, Nunc, ThermoFisher Scientific) coated with fibronectin at a density of 150,000 cells/cm^2^. When cells were ready for analysis, medium was removed, and wells were washed with 0.1 M phosphate buffer (PB) at RT for 2 min. Cells were fixed in 3% glutaraldehyde diluted in 0.1 M PB: first, glutaraldehyde was added and samples were incubated for at 37 °C 10 min. Fixative solution was removed and refreshed with new 3% glutaraldehyde solution, and samples were incubated at RT for 1 h. After that time, samples were washed with 0.1 M PB 5 times and stored at 4 °C. Post-fixation was carried out with 2% osmium tetroxide (OsO_4_) at RT for 1 h, and samples were stained in 2% uranyl acetate for 2 h at 4 °C protected from light. Samples were then rinsed in distilled water, dehydrated, and embedded in Durcupan ACM epoxy resin. Ultrathin sections (0.08 µm) were obtained with Ultracut UC-6 Ultramicrotome (Leica Microsystems, Wetzlar, Germany) and stained with Reynolds lead citrate. Images were captured on a FEI Tecnai Spirit BioTwin transmission electron microscope (ThermoFisher Scientific), equipped with a Xarosa digital camera (EMSIS GmbH, Münster, Germany). 

#### 2.7.2. Mitochondria Content Quantification

Analysis of the mitochondria morphology and content was performed using Zeiss Arivis Pro software (v.4.1.2) and FIJI (Image J v.2.14.0). Mitochondria were manually traced todetermine mitochondria surface area (µm^2^). Mitochondria content was determined by summing up mitochondria surface area per image. Data were collected from 40 images per condition, with a minimum of 10 mitochondria per image.

#### 2.7.3. Sarcomere Content Quantification

Z-band area was used as a surrogate for sarcomere content and quantified from electron micrographs using Zeiss Arivis Pro software by manual tracing.

### 2.8. Functional Analyses

#### 2.8.1. Calcium Imaging

iPSC C32-derived CMs were mounted on glass-bottom organ dishes (MatTek, P35G-1.5-14-c) coated with matrigel and incubated with Fluo-4 (Molecular Probes) at 5 µM for 30 min. Fluo-4 was washed in Tyrode’s solution for 30 min before imaging. Cells were placed in Tyrode’s solution containing CaCl_2_: 1.8 mM; NaCl: 130 mM; KCl: 5.4 mM; HEPES: 25 mM; MgCl_2_·6H_2_O: 0.5 mM; NaH_2_PO_4_: 0.4 mM; pH 7.4 at 37 °C for imaging. CMs were paced at 1 Hz and live imaging was recorded at 98 fps using a Nikon Eclipse with a Yokogawa CSU-X1 spinning disk and an EM-CCD Andor camera for high-speed acquisition. Individual whole-cell calcium transient intensities were analyzed in R software (V4.3.1). iCell CMs^2^ were plated in 96-well CellCarrier Ultra Phenoplates (PerkinElmer) coated with fibronectin and stained with the EarlyTox^TM^ Cardiotoxicity kit (Molecular Devices, San Jose, CA, USA), following the manufacturer’s recommendations. Calcium transients were analyzed in the FDSS/µCell system (Hamatsu Photonics, Hamamatsu, Japan). Data were analyzed using FDSS Wavefront Analysis Software (V.1.2.2E). Isoproterenol was used at a final concentration of 1 µM. Calcium transients are calculated as ∆F/F_0_, with signal leveling and background normalization, where F_0_ is background fluorescence and F is fluorescence intensity. For each cell, amplitude (peak intensity), upstroke velocity, decay velocity, and peak width at 70% duration (PWD70%) were calculated.

#### 2.8.2. Electrophysiological Analyses

For patch-clamp recordings, sterilized glass coverslips were coated with fibronectin and placed into each well of a 24-well plate, and 2 mL of iCell cardiomyoctes^2^ containing 50,000 cells was added to each coverslip for single-cell whole-cell recording and 100,000 for syncytial AP recording. When ready for analysis, cardiac APs and ionic currents were recorded with perforated (gramicidin) and ruptured whole-cell patch-clamp techniques, respectively. Coverslips containing plated hiPSC-CMs were transferred to an RC-26 recording chamber (Warner Instruments, Holliston, MA, USA) mounted onto the stage of an inverted microscope. Extracellular solution perfusion was continuous using a VC-6 valve controller (Warner Instruments). All recordings were performed at RT. Pipettes were pulled from thin-wall borosilicate glass capillaries with a PC-100 Vertical puller (Narishige, Tokyo, Japan) and had resistances between 1.8 and 3 MΩ with access resistances of <5 MΩ for ruptured patch recordings and 10–30 MΩ for gramicidin perforated patch recordings. Series resistance and cell capacitance were compensated to between 50 and 80% in all voltage-clamp recordings. Data were acquired at 10 or 100 kHz and filtered at 1 or 10 kHz with the Axon 700B Multiclamp, Digidata 1322A digitizer hardware and pClamp 11 software (Molecular Devices, San Jose, CA, USA) depending on experimental requirements. 

Cells were superfused with an extracellular solution containing (mM) NaCl 136.8, KCl 4, HEPES 10, CaCl_2_ 2, MgCl_2_ 1, and Glucose 10 (pH adjusted to 7.2 with NaOH) for AP recording and K^+^ current whole-cell recording. For whole-cell recording of Na^+^ and Ca^2+^ currents, the following extracellular solution was used (mM): NaCl 40, KCl 4, NMDG 96.8, HEPES 10, CaCl_2_ 2, MgCl_2_ 1, and Glucose 10 (pH adjusted to 7.2 with HCl); 1 mM of CdCl_2_ was also added to block L-type Ca^2+^ currents when recording Na^+^ currents.

Patch pipettes contained (mM): KCl 150, HEPES 10, EGTA 10, and MgATP 2 (pH adjusted to 7.2 with KOH). For AP recording, perforated patch recording was used, and pipettes were backfilled with a solution containing 50 μg/mL gramicidin (diluted from a 50 mg/mL gramicidin DMSO stock solution). A stock solution of ivabradine was prepared with DMSO. All stock solutions were diluted ≥1:1000 in extracellular solution before use. 

Spontaneous AP data analysis was performed using the CAPA software package (v3.40, SCCE, Essen, Germany). 

#### 2.8.3. Single-Cell Action Potential

SA121 hESC-CMs were seeded in 35 mm glass-bottom dishes (Ibidi) coated with fibronectin as described before, using a density of 50,000 cells/cm^2^. Cells were stained with FluoVolt (1:1000, Invitrogen, #F10488, Waltham, MA, USA) in a solution of cell culture medium containing pluronic acid, probenecid, and blebbistatin for 30 min at 37 °C and 5% CO_2_. After incubation, two washes with culture medium were performed and Tyrode’s solution was added for cell imaging in a Zeiss LSM 880 Airyscan microscope using a 20× objective. A 30 s video of each analyzed cell was recorded. Fluorescence intensity over time was analyzed using an R script (V4.3.1). Decrease in the slope curve (DSC), amplitude, and frequency of the obtained signals were measured and used to classify the cells with a ventricular, atrial, or pacemaker beating pattern.

### 2.9. Three-Dimensional Cardiac Microtissue Generation and Force Measurements

Prior to tissue formation, iCell cardiomyocytes^2^ were thawed and seeded in Plating Medium for 24 h, and primary cardiac fibroblasts (CC-2904, Lonza, Basel, Switzerland) were cultured in T75 flasks for up to one week. Cells were dislodged with TrypLE for 5 min at 37 °C and resuspended in 2 mg/mL collagen I, 0.8–0.95 mg/mL Matrigel, 1× MEM, 40 mM HEPESand 10% maintenance medium. Each tissue formation was initiated with 100,000 CMs and 10,000 cardiac fibroblasts in a 22 µL cell suspension which was added to Engineered Heart Tissue molds (µCTS, Novoheart, Boston, MA, USA). After two weeks in either Advanced RPMI/B27 or maturation medium, the contractile properties of the tissues were measured at a 1HZ frequency in Tyrode solution, by an automated force measurement system (CTScreen, Novoheart) that records the displacement of the PDMS pillars around which the tissues have formed, as described previously [38]. 

### 2.10. Cardiac Tissue Processing and Staining 

Cardiac tissues were fixed in 4% buffered formaldehyde solution for 24 h at RT. Fixed samples were stained with Mayer’s Hematoxylin Solution for 15 min at RT to facilitate tissue identification during cryosectioning, followed by embedding in cryomolds (Sakura, PN #4583) on dry ice and sample storage at −80 °C. Frozen tissue blocks were sectioned at a thickness of 10 µm using a Leica CM1950 cryostat, and slides were stored at −80 °C.

For immunohistochemistry, slides were incubated overnight at 4 °C with anti-α-sarcomeric actinin antibody (Abcam, PN #ab68167) diluted 1:500 in Dako Antibody Diluent (PN #S202230-2, Agilent, Santa Clara, CA, USA), followed by washes and incubation for 1 h at RT with Alexa-488 (ThermoFisher, #A-11008) or Alexa-565 (ThermoFisher, #A21201) secondary antibody at a dilution of 1:300 in antibody diluent. Nuclei were detected by incubating the slides with Hoechst 33342 solution (ThermoFisher, PN # 62249) at a dilution of 1:2000 in PBS-T for 10 min at RT. Stained slides were imaged with a Zeiss LSM 880 Airyscan microscope.

### 2.11. RNA Sequencing Analysis 

RNA extraction was performed using the Agencourt RNAdvance Cell V2 kit (catalog no. A47942, Beckman Coulter, Bromma, Sweden) according to the manufacturer’s instructions. RNA quality was assessed using the Fragment Analyzer Standard Sensitivity RNA kit from Agilent Technologies, ensuring an RNA integrity number greater than 7. Library preparation for iCell cardiomyoctes^2^ and Chips22 derived CMs was performed at the NGI (National Genomic Infrastructure, KTH, Stockholm, Sweden) using the Illumina TruSeq stranded mRNA library preparation kit. Clustering was conducted by ‘cBot’, and sequencing was performed on a NovaSeq 6000 (NovaSeq Control Software 1.6.0/RTA v3.4.4) with a 2 × 150 setup using the ‘NovaSeqXp’ workflow in the ‘S4’ flowcell. For the IPS C32-derived CMs, library preparation was carried out with the Illumina TruSeq Stranded Total RNA library preparation kit with Ribo-Zero Gold, following the manufacturer’s recommended protocols. This library was sequenced on a single lane of the Illumina HiSeq 3000 with a 2 × 150 setup using sequencing-by-synthesis chemistry v4, following the manufacturer’s guidelines. The Bcl-to-FastQ conversion was performed using bcl2fastq and the quality scale used was Sanger/phred33/Illumina 1.8+. Sequenced reads were quality-controlled with the FastQC software and pre-processed with Trim Galore. Processed reads from iCell cardiomyocytes^2^ and Chips22 were aligned to the reference genome GRCh37 with the STAR aligner, and Tophat2 with Bowties was used to map IPS C32 reads to GRCh38. Read counts for the genes were generated using the featureCounts library and normalized FPKM values calculated with StringTie. Read counts for the genes were generated using the featureCounts library and normalized FPKM values calculated with StringTie. Technical documentation on the RNA-seq pipeline is available here: https://github.com/nf-core/rnaseq (accessed on 8 April 2022).

### 2.12. Identification and Analysis of Differentially Expressed Genes

Differences in gene expression were identified using the DESeq2 method. The analysis was based on read counts, which are required to assess the measurement precision accurately. Separate analyses were performed for the iCell/ChiPS22 and iPSC C32 cell lines since they originated from separate experimental RNA-seq runs. Both analyses included sample categories in the DESeq2 design; in addition, the iCell/ChiPS22 analysis included a factor for batch correction. Differentially expressed genes (DEGs) were reported considering a FDR < 0.05 and |log2 FC| > 1. For PCA analysis, heatmaps, and other statistical plots, the data were transformed using the variance stabilizing transformation (VST) functionality included in the DESeq2 R package (v1.36.0). According to the DESeq2 documentation, this is similar to transforming the data to a log2 scale. Volcano plots were created using R (v. 4.2.3) and ggplot (v.3.5.0).

PCA analysis was performed using the PCATools R package (v2.8.0), and the ComplexHeatmap R package (v2.12.1) was used for heatmap generation [39]. 

### 2.13. Pathway Enrichment Analysis

The DEGs were used for enrichment analysis using IPA (Ingenuity Pathways Analysis; QIAGEN Inc., version 111725566, Germantown, MD, USA). This tool uses the information in the Ingenuity^®^ Knowledge Base to assess signaling and metabolic pathways, upstream regulators, regulatory effect networks, and disease and biological functions that are likely to be perturbed based on a dataset of interest (in our case, the DEGs). The IPA upstream analysis was performed to identify significantly dysregulated canonical pathways and biological functions in standard versus maturation medium and between the ET-1-treated and non-treated groups. The enrichment was tested using Fisher’s exact test with adjusted *p*-value < 0.05. We used a |Z-score| ≥ 1.5 to consider functions/pathway activation or inhibition. 

Additionally, we performed an overrepresentation analysis of DEGs of each cell line and for overlapping genes using the KEGG database and the clusterProfiler R package (version 4.6.2) [40]. Significant pathways were selected with an adjusted *p*-value < 0.05. All graphs were created using R v. 4.2.3 and ggplot v.3.5.0.

### 2.14. Isolation of Total RNA from Cell Cultures and Quantitative Real-Time PCR (qPCR)

Total RNA was isolated using a Qiagen RNeasy kit on a Qiacube automatic system, and RNA concentration and quality were measured using a spectrophotometer (Nanodrop 2000, ThermoFisher). cDNA strands were synthesized through a reverse transcription reaction in a Thermocycler using a QuantiTect Reverse Transcription Kit following the manufacturer’s instructions. Amplification and fluorescent quantification were obtained from an ABI QuantiStudio 5 Real-Time PCR System (ThermoFisher). The following TaqMan^TM^ probes from ThermoFisher were used: MYL2 (#Hs00166405_m1), MYH6 (#Hs01101425), MYH7 (#Hs01110632_m1), TNN3 (#Hs00952980) TNN1 (#Hs00913333_m1), and RPLP0 (#Hs00420895) as a housekeeping gene. Relative quantification of target gene expression was performed using ddCt method.

### 2.15. In Vitro-to-In Vivo Translatability of BCKDK Inhibition on BCAA Catabolism

BCKDK inhibitors were dosed at 8 concentrations (1 µM–0.01 µM) on cells cultured in either standard or maturation medium for 19 days as described above. The dosing medium contained no ACC2i. After 24 h, the plate was centrifuged for 1 min at 3200× *g* and incubated with Internal Standard Solution (4 µM ketovaline-C13C5 in acetonitrile and methanol at a ratio of 1:1) until cells were lysed. Equal volumes of the cell lysates and 0.1% formic acid were added to 384 well plates, centrifuged for 1 min at 3200× *g,* and injected into an API-5000 mass spectrometer (Sciex) via a Symmetry C8 column (Waters).

All animal studies were performed according to AstraZeneca’s Institutional Animal Care and Use Committee guidelines (ethical approval number 5833-2023) using methods for compound dosing and plasma analysis as previously described [41,42]. Briefly, healthy male Sprague Dawley rats were treated for 24 h with vehicle only, or a single dose of BCKDK inhibitor. Samples for exposure and keto acid analyses were taken 0, 0.5, 1, 3, 5, 7, and 24 h after dosing. The longitudinal data were modeled using a turnover model with an inhibitory function on the synthesis rate and an in vivo IC50 was estimated. The unbound in vivo IC50s were used in the correlation plot. Al modeling was performed using Phoenix NMLE 8.3 (Certara L.P, Radnor, PA, USA). BCKA in rat plasma was measured using ultra-high-performance liquid chromatography coupled to a Waters TQ-XS triple quadrupole (UPLC-MS/MS). Pearson correlation analysis was used to evaluate the correlation between in vitro and in vivo IC50s for ketovaline concentration.

### 2.16. Statistical Analysis

Statistical analysis was performed using GraphPad Prism Software (v.9.5.1, Dotmatics, Boston, MA, USA). Values are represented as mean ± SD or as mean ± SEM of independent measurements or assays. Statistical significance was evaluated using either an unpaired Student’s *t*-test or one-way analysis of variance (ANOVA), unless otherwise stated. *p*-values < 0.05 were considered statistically significant.

## 3. Results

Aiming to develop an enhanced maturation medium formulation, we investigated the efficacy of ACC2 inhibition in combination with specific metabolic conditioning and biochemical cues. Specifically, the medium is based on glucose-depleted basal medium, supplemented with CP640186, a potent ACC inhibitor [43], FAs, and galactose to promote FAO, a key metabolic pathway in mature CMs, and a TID cocktail, comprising triiodothyronine hormone (T3), insulin-like growth factor 1 (IGF-1), and synthetic glucocorticoid dexamethasone (Dex), shown previously to enhance hPSC-CM contractility and functionality [5]. This medium composition is here referred to as maturation medium. We evaluated the efficacy of this novel maturation medium on hPSC-CMs derived from four different cell lines (three hiPSC lines and one hESC line) at various stages of differentiation (between 15 and 35 days). Our assessment focused on several key CM maturation features: (i) improved oxidative metabolism/bioenergetics; (ii) induction of adult-like CM gene expression profile; (iii) enhanced calcium signaling, contractile function, and electrophysiology; (iv) developed and organized cell structure and ultrastructure; (v) disease modeling capabilities and enhanced translatability. For comparison, we cultured hPSC-CMs in either the presented maturation medium or in their corresponding standard maintenance medium for the same time period. 

### 3.1. Enhancement of Mitochondrial Function, FAO, and Bioenergetics in hiPSC-CMs Cultured in Maturation Medium Containing an ACC2 Inhibitor

To elucidate the potential of ACC inhibition in improving hiPSC-CM metabolism, we focused on CP-640186, known for its robust inhibition of ACC across various cell and animal models [43]. CP-640186 effectively targets both ACC isoforms (ACC1 and ACC2) with IC50 values of approximately 55 nM, and thereby decreases malonyl-CoA levels, enhances CPT-1 activity, inhibits FA biosynthesis, and stimulates FAO (Figure S1A) [43].

We initially investigated the impact of increasing concentrations of the ACC inhibitor CP640186 (hereafter referred to as ACC2i, given the predominant expression of ACC2 in CMs) on hiPSC-CM viability. Our results revealed that ACC2i concentrations exceeding 5 uM resulted in a decrease in the number of nuclei, indicating potential cardiac toxicity at higher doses of ACC2i (Figure S1B). Based on these initial results, a concentration of 5 uM was selected for further examination of the role of ACC2i in improving hiPSC-CM maturation.

**Figure 1:**
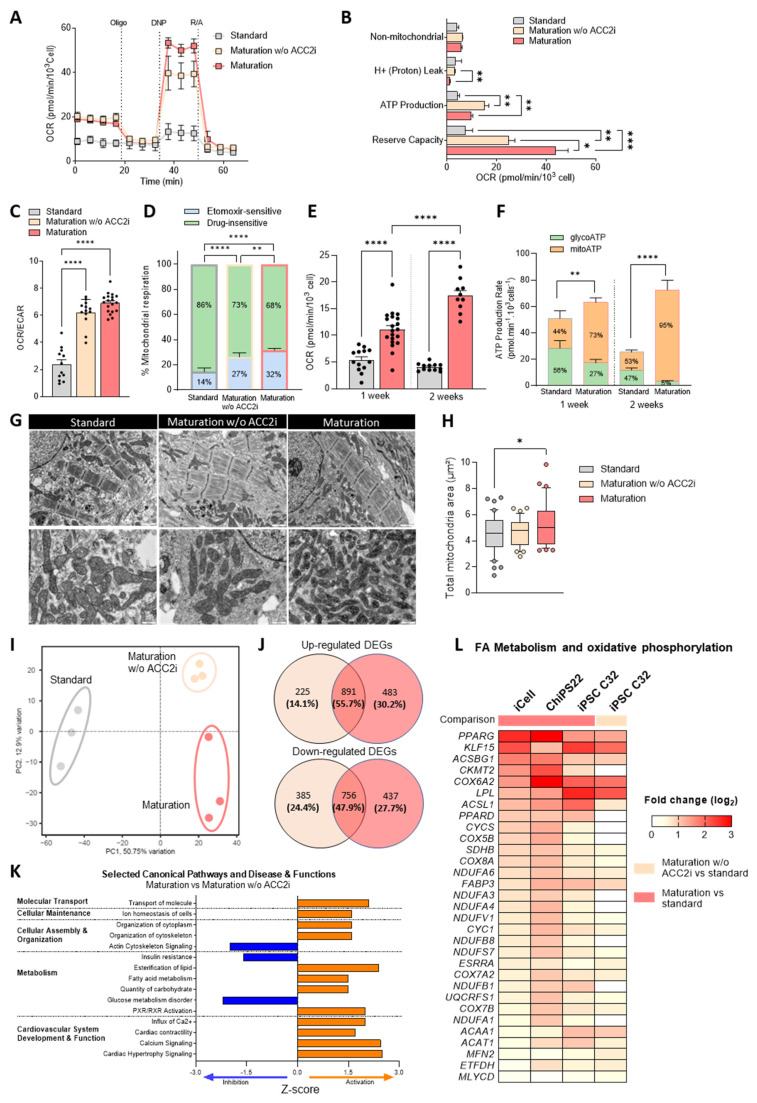
Maturation medium improves mitochondrial function and oxidative metabolism of hiPSC-CMs. (**A**–**F**) Bioenergetics measurements of hiPSC-CMs cultured in standard (grey), maturation medium (red) and maturation medium without (w/o) ACC2i (orange) by Seahorse extracellular flux analyzer (*n* = 3–4 batches per group). (**A**) Representative kinetics of the oxygen consumption rate (OCR) during mitochondrial stress test. Cells were treated with the ATP synthase inhibitor Oligomycin (Oligo), the respiratory uncoupler (DNP), and the respiratory chain blockers rotenone and antimycin A (R/A). (**B**) Proportion of OCR due to non-mitochondrial oxygen consumption, proton leak, ATP production, and reserve capacity. (**C**) OCR/ECAR ratio. (**D**) Sensitivity of basal mitochondrial respiration to etomoxir as a measurement of % of OCR related to FA oxidation. (**E**,**F**) Comparison of basal OCR (**E**), mitochondrial ATP production, and glycolytic ATP production (**F**) after one and two weeks in culture in standard vs. maturation medium. (**G**) Representative TEM images of mitochondria in hiPSC-CMs cultured in standard medium, maturation medium, and maturation medium w/o ACC2i. Scale bars: 1 µm and 500 nm. (**H**) Quantification of mitochondrial content using total mitochondria area per TEM image. Data collected from 40 images per condition, with a minimum of 10 mitochondria per image. (**I**) Two-dimensional principal component analysis using RNA-seq data of hiPSC-CMs cultured in standard, maturation, and maturation medium w/o ACC2i. (**J**) Venn diagrams of overlapping DEGs (FDR < 0.05 and |log2 FC| > 1). (**K**) Significantly activated canonical pathways and disease functions associated with CM development, function, and metabolism comparing maturation medium vs. maturation medium w/o ACC2i using IPA analysis. (**L**) Heatmap depicting changes in the RNA-seq expression of FAO and oxidative phosphorylation-related genes across three hiPSC lines, comparing maturation or maturation w/o ACC2i vs. standard medium. Color scale shows only positive values since only upregulation was observed for these genes. Statistical analyses were performed by one-way ANOVA with Tukey’s multiple comparisons test in A-H. Data are presented as mean ± SEM. * *p* < 0.05, ** *p* < 0.01, *** *p* < 0.001, and **** *p* < 0.0001.

We compared the metabolic and bioenergetic profiles of hiPSC-CMs cultured in maturation medium, maturation medium without ACC2i, and standard culture medium, using the Seahorse analyzer (Figure 1A–F). HiPSC-CMs cultured in maturation medium exhibited a highly energetic phenotype when compared to hiPSC-CMs cultured in standard medium, as evidenced by a significant increase in oxygen consumption rate (OCR; 19.8 vs. 8.2 pmol/min/10^3^ cell (*p* = 0.0007), last baseline measurement before Oligomycin injection; Figure 1A), reserve capacity (Figure 1A,B), and OCR/extracellular acidification rate (ECAR) ratio (Figure 1C). These cells also showed a 2.3-fold increased reliance on FAO (Figure 1D), compared to cells cultured in standard medium. 

Notably, the addition of ACC2i further enhanced bioenergetic efficiency. A significant 1.7-fold increase in reserve capacity and a marked 2.5-fold reduction in energy dissipation through proton leak was observed when comparing maturation medium versus maturation medium without ACC2i (Figure 1B). ACC2 inhibition also significantly increased FAO reliance, as demonstrated by a significant change in basal respiration rate upon inhibition of FA uptake into mitochondria by the CPT1 inhibitor, etomoxir (Figure 1D). 

Extended culture in maturation medium for two weeks resulted in significantly higher basal respiration (Figure 1E) and mitochondrial ATP production (Figure 1F) compared to one-week cultures, leading us to conduct subsequent analyses with a two-week culture period. Upon comparison with isolated adult mouse CMs, which inherently exhibit elevated OCR and ATP production to meet the demands of a higher heartbeat rate and resulting increased energy requirements, hiPSC-CMs cultured in maturation medium demonstrated comparable percentages of glycolytic and mitochondrial ATP production (Figure S2).

In addition, transmission electron microscopy (TEM) analysis showed increased mitochondrial content positioned closer to the sarcomeres in cells cultured in maturation medium (Figure 1G,H). 

Consistent improvements in mitochondrial substrate oxidation and an increase in mitochondrial content were also observed in CMs derived from an additional hiPSC line (Figure S3A–C), underscoring the maturation medium’s effectiveness in promoting mitochondrial bioenergetics. 

To uncover the mechanisms underlying the observed effects, we performed RNA sequencing on CMs derived from three different hiPSC lines (Figure 1I–L and Figure S4). The transcriptional signature significantly differed between cells cultured in maturation versus standard medium (Figure 1I and Figure S4A–D). A large number of up- and downregulated genes were identified in all three cell lines (Figure S4B–D). Additional distinctions were observed when comparing cells in maturation medium with cells cultured in maturation medium without ACC2i, as evidenced by a clear group separation in the principal component analysis (Figure 1I). Cells cultured in maturation medium exhibited a slightly higher number of differentially expressed genes (DEGs: FDR < 0.05 and |log2 FC| > 1) than in the absence of ACC2i (2567 vs. 2257, respectively) with only approximately 50% overlapping DEGs (Figure 1J). A comparison between these two groups using Ingenuity Pathway Analysis showed a significant differential regulation in pathways and biological functions related to molecular transport, cellular maintenance, assembly and organization, metabolism, and cardiovascular function (Figure 1K). In particular, ACC2i significantly activated FA metabolism but also cardiac contractility, calcium signaling, and cardiac hypertrophy pathways (Figure 1K), suggesting that ACC2i may also contribute to cardiac functional and structural changes. Accordingly, when comparing significant enriched pathways in maturation versus standard medium, we observed similar changes in cardiac function- and metabolism-related pathways in all three hiPSC lines, such as the calcium signaling pathway, cardiac muscle contraction, oxidative phosphorylation, FA metabolism, the PPAR signaling pathway, and steroid biosynthesis (Figure S4E–G). Additionally, the three hiPSC lines cultured in maturation medium exhibited a consistent upregulation of FA and oxidative metabolism-related genes (*CKMT2*, *COX6A2*, *LPL*, *ACSL1*, *FABP3*, *ACAT1*), as well as mitochondrial genes associated with the electron transport chain (including NADH-ubiquinone oxidoreductase chain, cytochrome bc complex, and cytochrome c oxidase subunits) (Figure 1L). The expression of these genes did not change or increase at lower levels in maturation medium without ACC2i. Importantly, the expression of key regulators of the PPAR (peroxisome proliferator-activated receptor) family, particularly *PPARD* expression, was significantly increased in hiPSC-CMs cultured in maturation medium but not in hiPSC-CMs cultured in maturation medium without ACC2i (Figure 1L). *KLF15* (Krüppel-like factor 15), a zinc-finger transcription factor playing a critical role in cardiac lipid utilization [44], was also significantly upregulated in all hiPSC lines, and its expression was further enhanced by ACC2i (Figure 1L). 

In summary, our results demonstrate that ACC2i significantly enhances mitochondrial efficiency and content, substrate oxidation, and bioenergetics in hiPSC-CMs. These effects were further supported by associated transcriptional changes, suggesting that the presented medium formulation can effectively improve hiPSC-CM metabolic maturation.

### 3.2. Maturation Medium Enhances Calcium Kinetics in hiPSC-CMs

**Figure 2:**
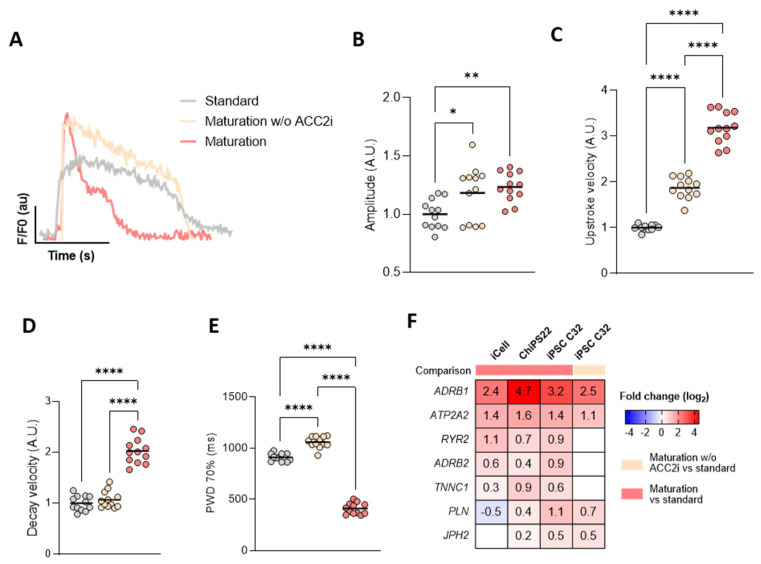
Maturation medium enhances Ca^2+^ handling properties of hiPSC-CMs**.** (**A**) Representative Ca^2+^ transients recorded by EarlyTox Cardiotoxicity calcium dye for iCell CMs^2^. Quantification of Ca^2+^ transient amplitude (**B**), upstroke velocity (**C**)**,** decay velocity (**D**), and peak width at 70% duration (PWD 70%) (**E**). Each tracer is an average normalized change in fluorescence (F − F_0_)/F_0_ versus time plot from multiple peaks, where F means fluorescence intensity and F_0_ basal fluorescence. Panels B–D are represented as fold change using the standard medium experimental group as a reference. (**F**) Fold change in expression of sarcoplasmic reticulum (SR) genes involved in Ca^2+^ handling and beta-adrenergic receptors depicting a general increase in hiPSC-CMs cultured in maturation medium with greater differences in the hiPSC C32 cell line. Only statistically significant genes with FDR < 0.05 and |log2 FC| > 0.2 are represented. ATP2A2 or SERCA2a, sarco/endoplasmic reticulum Ca^2+^-ATPase; PLN, phospholamban; RYR2, ryanodine receptor 2; JPH2, junctophilin 2. Statistical analyses were performed by one-way ANOVA with Tukey’s multiple comparisons. Data are presented as mean ± SEM. * *p* < 0.05, ** *p* < 0.01, and **** *p* < 0.0001. Colors in all panels correspond to standard (grey), maturation medium w/o ACC2i (orange), and maturation medium (red).

To investigate whether the maturation medium, specifically ACC2 inhibition, enhances cardiac function, we quantified Ca^2+^ dynamics using a calcium fluorescent dye (Figure 2). 

Cultivation of hiPSC-CMs in maturation medium resulted in a marked increase in CM calcium transient amplitude and upstroke and decay velocities, and a shorter calcium transient duration compared to hiPSC-CMs maintained in standard medium (Figure 2A–E). When comparing iCell CMs cultured in maturation medium with iCell CMs cultured in maturation medium without ACC2i, we observed a significant enhancement in calcium uptake and decay kinetics and a reduction in transient duration, but no change in transient calcium amplitude (Figure 2B–E). In iPSC C32 CMs, both the calcium transient amplitude and kinetics were significantly improved with ACC2i (Figure S5). 

In agreement with the improved calcium kinetics observed in matured hiPSC-CMs, increased expression levels of genes related to sarcoplasmic reticulum (SR) Ca^2+^ handling, specifically the SR Ca^2+^-ATPase (*ATP2A2;* SERCA2a) and the ryanodine receptor 2 (*RYR2*), were observed across all cell lines cultured in maturation medium (Figure 2E). *PLN* (a critical regulator of SERCA2a) and junctophilin 2 (*JPH2*, a key player in cardiac excitation–contraction coupling) were more pronouncedly upregulated in iPSC C32 CMs (Figure 2E). The upregulation of SERCA2 and PLN in iPSC C32 CMs was also confirmed at the protein level by immunofluorescence staining (Figure S5E). The variation observed in differential gene expression may reflect the hiPSC line-dependent variability of their maturation status due to intrinsic differences regarding their differentiation capacity as well as differences in culture time before switching their culture to maturation medium.

Irrespective of the culture duration, differentiation method, or intrinsic maturation status, our results suggest that ACC2 inhibition consistently improves Ca^2+^ handling. 

Upon stimulation with isoproterenol, cells cultured in maturation medium showed a significantly higher increase in beat rate and amplitude, compared to cells cultured in maturation medium without ACC2i (Figure S5A–D), indicative of either a more effective and robust β-adrenergic responsiveness, or the presence of an increased cardiac reserve, or both. In agreement, ADRB1 and ADRB2 genes encoding for β-adrenergic receptors are significantly upregulated (Figure 2E) and the “adrenergic signaling in cardiomyocytes” pathway significantly enriched (Figure S4E,G) in hiPSC-CMs cultured in maturation medium. 

### 3.3. Superior Electrophysiological Properties of hiPSC-CMs Cultured in Maturation Medium Compared to Standard Medium 

**Figure 3:**
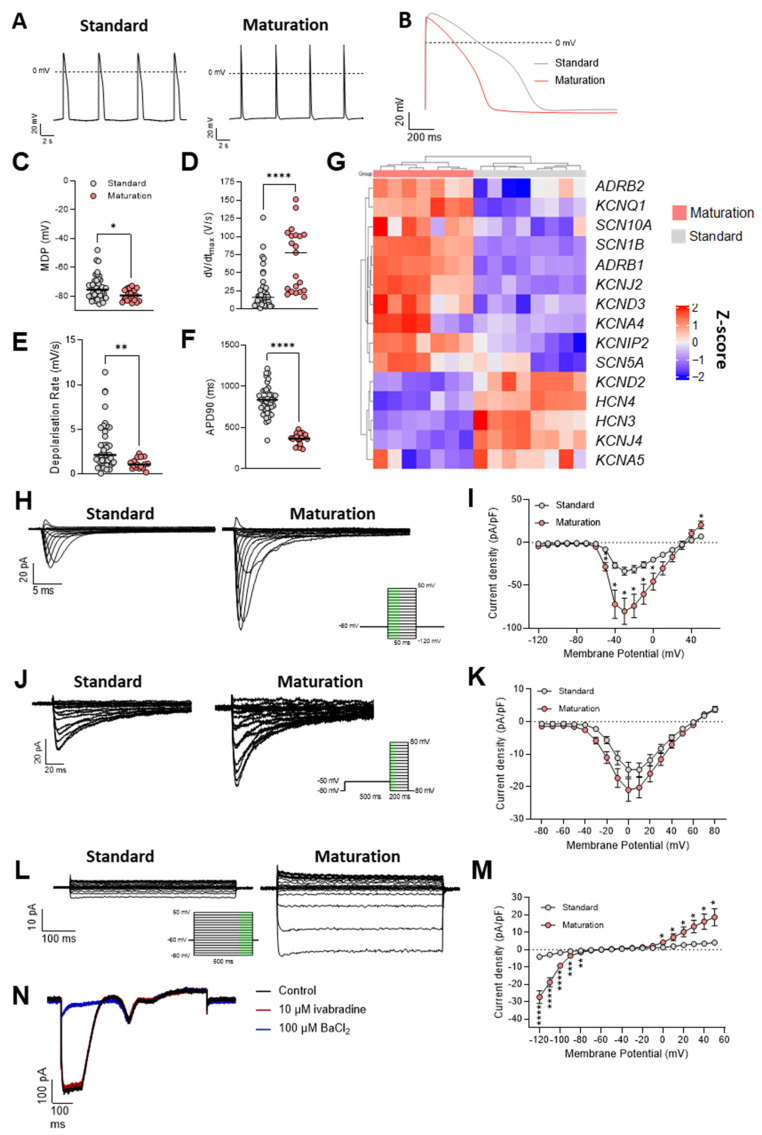
HiPSC—CMs cultured in maturation medium preserve a ventricular phenotype and show more mature electrophysiological properties. Patch-clamp analysis of iCell CMs^2^ cultured in standard medium (grey) and maturation medium (red) showing improved diastolic membrane potential, upstroke velocity, and inward rectifier (I_K1_) current in maturation medium. (**A**,**B**) Representative action potential (AP) traces from single spontaneously beating hiPSC-CMs. (**C**) Maximum diastolic potential (MDP). (**D**) AP upstroke velocity (dV/dtmax). (**E**) Depolarization rate. (**F**) AP duration at 90% repolarization. (**G**) Heatmap representing expression levels of key cardiac ion channels in hiPSC-CMs cultured in both standard and maturation media. (**H**) Representative Na^+^ current (I_Na_) peak. Inset: voltage − clamp protocol. (**I**) Current–voltage relationship for I_Na_ (*n* = 17 maturation medium, *n* = 12 standard medium). (**J**) Representative Ca^2+^ current (I_Ca,L_) peak. Inset: voltage-clamp protocol. (**K**) Current–voltage relationship for I_Ca,L_ *(n* = 15 maturation medium, *n* = 9 standard media). (**L**) Large inward rectifier K^+^ current (I_K1_). Inset: voltage-clamp protocol. (**M**) Current–voltage relationship for I_K1_ (*n* = 18 maturation medium, *n* = 14 standard medium). (**N**) Representative I_K1_ traces in control and following the application of 100 µM BaCl_2_ and 10 µM ivabradine. Outward components of Ba^2+^ − sensitive I_K1_ and voltage protocol are given in insets. Statistical analyses were performed by unpaired Student’s *t*-tests relative to standard medium. Data are presented as mean ± SEM. * *p* < 0.05, ** *p* < 0.01, *** *p* < 0.001, and **** *p* < 0.0001.

To further investigate the impact of the described maturation medium on hiPSC-CM function, we conducted a comprehensive electrophysiological analysis (Figure 3). Analysis of action potentials (APs) revealed distinct differences in AP morphology between hiPSC-CMs cultured in maturation versus standard media (Figure 3A,B, Table S1). hiPSC-CMs in maturation medium exhibited a significantly more negative maximum diastolic potential (MDP) of −79.2 ± 4 mV vs. −74.3 ± 8.2 mV (Figure 3C), an increase in the maximal rate of depolarization (dV/dt max) of 68.6 ± 43.5 V/s vs. 23.4 ± 25.6 V/s (Figure 3D), a decreased diastolic depolarization rate of 1 ± 0.7 mV/s vs. 3.2 ± 2.8 mV/s (Figure 3E), and a reduced AP duration (APD90) of 357 ± 69.6 ms vs. 828.8 ± 176.1 ms (Figure 3F). Altogether, these changes strongly suggest an enhanced electrophysiological maturity of hiPSC-CMs when cultured in the maturation medium. To elucidate the ionic currents underlying these observed differences in AP morphology, voltage-clamp experiments were performed.

hiPSC-CMs in maturation medium demonstrated a 2-fold increase in sodium current (I_Na_, Figure 3H,I), contributing to the observed faster dV/dt max in the AP (Figure 3D). The peak I_Na_ (−30 mV), normalized to cell capacitance, was significantly higher in the maturation group (−80.4 ± 61.8 pA/pF) compared to controls (−33.3 ± 15.2 pA/pF), supporting the evidence of more mature hiPSC-CMs. In adult CMs, Na^+^ is utilized to generate the AP, whereas immature hiPSC-CMs often rely on Ca^2+^ for inward currents to produce electrical responses.

The calcium (I_Ca,L_) current, crucial for the AP plateau phase and muscle contraction, increased by approximately 40% in the maturation group (Figure 3J,K). The peak I_Ca,L_ (0 mV), normalized to cell capacitance, was −21.0 ± 12.9 pA/pF in the maturation medium group compared to −14.7 ± 6.8 pA/pF in the standard medium group. 

**Figure 4:**
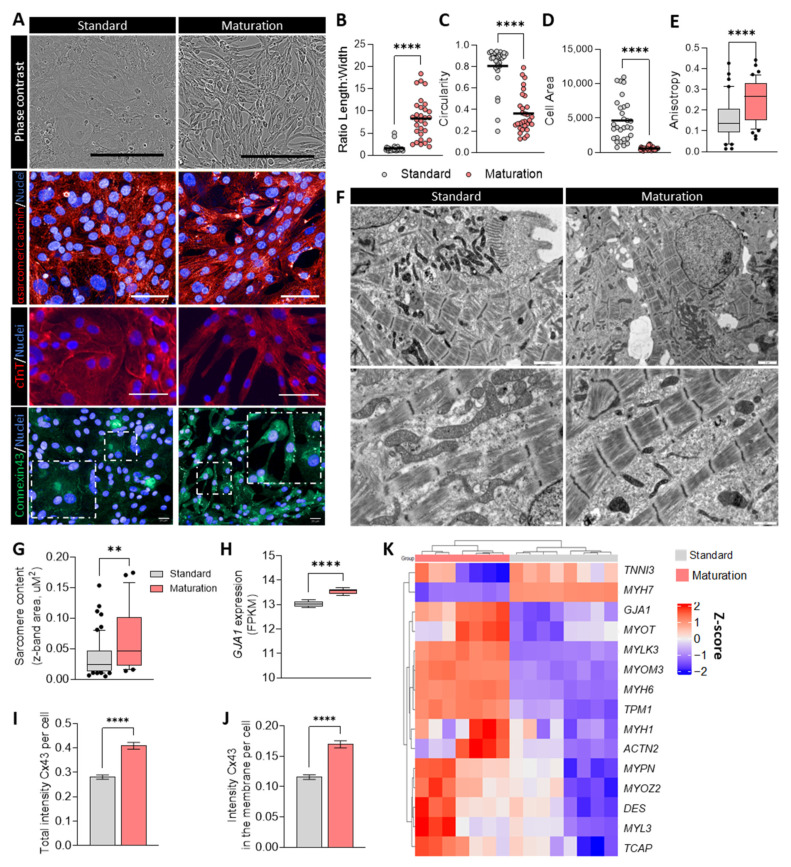
Maturation medium induces morphological and ultrastructural maturation in hiPSC-CMs. (**A**) Representative phase contrast and confocal fluorescent images of α-sarcomeric actinin, cardiac troponin T (cTnT) and connexin 43 staining in cells cultured in standard and maturation medium. Scale bars: 100 µm (phase contrast), 50 µm (α-sarcomeric actinin and cTnT), and 20 µm (connexin 43). (**B**–**D**) Cell structure characterization in terms of length-to-width ratio (**B**), circularity index (**C**), and cell area (**D**). (**E**) Quantification of sarcomere anisotropy as a measurement of alignment based on α-sarcomeric actinin staining. (**F**) Representative TEM images showing more organized and aligned sarcomere structures of hiPSC-CMs cultured in maturation medium vs. standard medium. Scale bars: 2 µM (top row) and 500 nm (bottom row). (**G**) Sarcomere content based on quantification of Z-band area in TEM images. (**H**) Expression levels of *GJA1* gene (coding connexin 43 (Cx43) protein). Comparison of the total intensity of Cx43 immunostaining per cell (**I**) and intensity of Cx43 co-localized in the plasma membrane per cell. The plasma membrane was visualized using CellBrite membrane dye (**J**). (**K**) Heatmap depicting expression levels of sarcomere genes. Statistical analyses were performed by unpaired Student’s *t*-tests relative to standard medium. Data are presented as mean ± SEM., ** *p* < 0.01, and **** *p* < 0.0001.

Furthermore, both inward rectifier K^+^ current (I_K1_) and outward K^+^ currents, essential for the repolarization of the cardiac AP, were significantly enhanced in maturation medium (Figure 4L,M). Remarkably, I_K1_ exhibited a 6-fold increase, emphasizing its key role in setting a more negative MDP. The peak I_K1_ (−120 mV) was −27.3 ± 2.6 pA/pF in the maturation group versus −4.1 ± 2.6 pA/pF in standard medium group. The peak outward K^+^ current density (60 mV) increased 1.4-fold in maturation medium (−21.0 ± 12.9 pA/pF vs. −14.7 ± 15.4 pA/pF). Additionally, the inward current in hiPSC-CMs cultured in the maturation medium was shown to be insensitive to 10 µM ivabradine (~5% block) but fully blocked by 100 µM Ba^2+^ (>90% block) (Figure 3N), confirming that the measured inward current is I_K1_, encoded by Kir channels. The lack of response to ivabradine, a well-known inhibitor of the pacemaker “funny” (If) channel, also suggests the absence of I_f_ current in these cells. In agreement, increased I_K1_ and outward K^+^ currents and reduced I_f_ are all hallmarks of improved CM maturation. The improvement in cardiac response was also demonstrated through the impact of dofetilide, a specific hERG blocker, on the AP of hiPSC-CMs following maturation (Figure S6). Previous research [45] has indicated that the relative immaturity of hiPSC-CMs, particularly the absence of the I_K1_ current, led to hERG blockers reducing the maximum diastolic potential (MDP), which subsequently could decrease the maximum depolarization rate (MDR) through the inactivation of Na^+^ channels. However, after maturation, dofetilide did not significantly affect MDP and MDR, but it did result in a 25% prolongation of the APD90 (Figure S6A,B), indicating an enhancement in the CM response to the blockers.

In accordance with the improved electrophysiological properties, hiPSC-CMs cultured in maturation medium also showed significant increases in the expression of sodium and potassium channels compared to those in standard medium (Figure 3G and Figure S3D). Specifically, maturation medium enhanced the expression of *SCN5A* and *SCN1B* (sodium channels critical for the upstroke velocity of the AP), *KCNQ1* and *KCNJ2* (potassium channels vital for I_K1_ current density), and *KCNIP2* (modulator of the Kv4 potassium channel family affecting the fast transient outward potassium current (I_to,f_) and early cardiac repolarization). Conversely, expressions of *HCN4* and *HCN3*, linked to automaticity, were reduced (Figure 3G and Figure S3D), aligning with gene expression characteristics of adult ventricular CMs.

### 3.4. Improved Phenotypic Maturation of hiPSC-CMs Cultured in Maturation Medium

In addition to improved metabolism and cardiac function, hiPSC-CMs cultured in maturation medium also showed improved phenotypic and morphologic characteristics when compared to cells cultured in standard medium. Phase contrast microscopy and co-staining for α-sarcomeric actinin and troponin T revealed enhanced cell alignment and sarcomere organization in hiPSC-CMs cultured in maturation medium (Figure 4A). Furthermore, hiPSC-CMs in maturation medium demonstrated a significant 5-fold increase in aspect ratio (length/width ratio, Figure 4B), indicating a more elongated morphology. This finding was accompanied by a significant reduction in circularity (Figure 4C) and cell area (Figure 4D), as well as a 1.6-fold increase in anisotropy compared to cells cultured in standard medium (Figure 4E). These morphological changes suggest a shift towards a more mature CM phenotype, characterized by elongated, rod-shaped, and anisotropic cells, important to enhance electrical conduction and contractile efficiency.

TEM analysis further confirmed a more mature sarcomere structure and higher sarcomere content in hiPSC-CMs cultured in maturation medium (Figure 4F,G). These cells exhibited well-defined Z-lines as well as A- and I-bands, with a higher density of aligned myofibrillar structures across the entire CM area, in contrast to the less dense, randomly aligned myofibrils observed in standard medium cultured hiPSC-CMs (Figure S7A). Additionally, the presence of T-tubules and a higher abundance of intercalated disk-like structures and desmosomes at the transverse boundaries between neighboring CMs were noted in the maturation medium group (Figure S7B).

Importantly, maturation medium significantly enhanced the transcription, protein expression, and polarization (around the cell membrane) of connexin 43 (Figure 4A,H–J), a key feature of mature CMs. 

Myofibrillar structural genes were also upregulated in maturation medium (Figure 4K), particularly Z-disk genes, such as *DES*, *MYOT*, *MYPN*, *MYOZ2*, and *TCAP*, which are implicated in coordinating sarcomeric organization. Consistent with previously reported findings, hiPSC-CMs in maturation medium displayed a decrease in *MYH7* expression and an increase in *MYH6* expression, leading to an elevated MYH6-to-MYH7 ratio. This shift, although opposite to what is observed in adult CMs, is indicative of a certain level of cellular physiological hypertrophy and is known to be induced by T3 treatment [3,5]. 

The observed changes in cell morphology, organization, ultrastructure, and expression of structural genes were consistent across different hiPSC lines (Figure S3E–I), underscoring the robustness of the maturation medium’s effects at the cell structure level.

### 3.5. Maturation Medium Enhances Cell Structure, CM Marker Expression, and Ventricular Phenotype in hESC-CMs

Comparable improvements in morphology and transcriptional changes were also obtained with hESC-CMs cultured in maturation medium versus standard medium (Figure S8A,B). Single-cell action potential analysis revealed a significant enrichment of the ventricular subpopulation in hESC-CMs cultured in maturation medium, with an increase from 60% to 80% compared to standard medium. This shift was accompanied by a decrease in the proportion of atrial and pacemaker cell phenotypes (Figure S8C). These findings underscore the versatility and effectiveness of the presented maturation medium, demonstrating its potential to enhance maturation of both hiPSC-CMs and hESC-CMs.

### 3.6. Maturation Medium Improves Formation, Morphology, and Contractile Kinetics of hiPSC-CM-Derived 3D Microtissues

**Figure 5:**
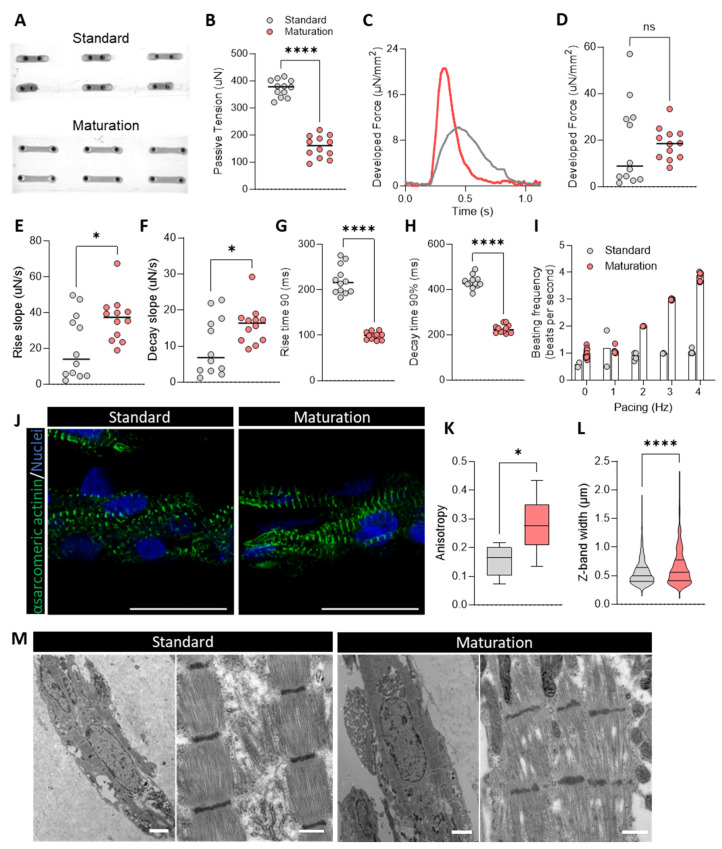
Maturation medium enhances contractile kinetics and structural integrity of hiPSC-CM 3D microtissues. Contractile force and kinetics measured using the Novoheart microtissue platform depict faster contractile kinetics and improved structural integrity in microtissues cultured in maturation medium. (**A**) Representative brightfield images of cardiac microtissues two weeks after culture in standard and maturation medium. (**B**) Passive tension measured at the last day of culture. (**C**) Representative contractility traces of cardiac microtissues paced at 1 Hz. (**D**) Quantification of contraction force. (**E**) Rising slope as a measurement of upstroke velocity. (**F**) Decay slope as a measurement of relaxation velocity. (**G**,**H**) Time to 90% rise and 90% decay, respectively. All measurements shown in C–H were acquired from microtissues paced at 1 HZ in Tyrode solution to provide comparable conditions. (**I**) Pacing frequency–beating frequency relationship showing that microtissues in maturation medium follow a short-term increase in pacing frequency in contrast to microtissues in standard medium. Microtissues were paced up to 4 Hz with increments of 1 HZ every 30 s. (**J**) Representative confocal fluorescent images of α-sarcomeric actinin in microtissues cultured for two weeks in standard and maturation medium. Scale bars: 25 µM. (**K**) Quantification of sarcomere anisotropy as a measurement of alignment based on α-sarcomeric actinin staining. (**L**) Quantification of sarcomere width as a measurement of myofibrillar density based on α-sarcomeric actinin staining. (**M**) Representative electron microscopy images of micro tissues cultured in either standard or maturation medium highlighting increased sarcomere and mitochondria content with maturation medium. Scale bars: 2 µM and 500 nm. Statistical analyses were performed by unpaired Student’s *t*-tests using standard medium as reference or one-way ANOVA with Tukey’s multiple comparisons. *n* = 12 microtissues were used. Data are presented as mean ± SEM. * *p* < 0.05, and **** *p* < 0.0001.

Next, we explored the effect of the maturation medium in 3D microtissues, which represent a physiologically more relevant in vitro model to assess cardiac contraction and relaxation properties. Microtissues were formed around flexible pillars in the µCTScreen platform (Novoheart), which allows electrical stimulation of the microtissues, as well as functional measurements. After two weeks of culture in maturation medium, microtissues exhibited superior structural integrity, maintenance of a consistent tissue width and viability over time (Figure 5A). In contrast, microtissues cultured in standard medium often lost their structural integrity, forming clumps or aggregates, as reflected by an increased passive tension indicating more aggregated tissues (Figure 5B).

Microtissues cultured in maturation medium showed a trend towards increased developed force in comparison to those in standard medium, although variability in the latter group precluded statistical significance (Figure 5C,D). Notably, microtissues in the maturation medium demonstrated significantly enhanced contraction and relaxation kinetics, as evidenced by increased rising and decay slopes, and reduced contraction and relaxation times (Figure 5E–H). Furthermore, these tissues maintained responsiveness to high-frequency field stimulations up to 4 Hz, a strong contrast to tissues in standard medium, which failed to follow pacing beyond 1 Hz (Figure 5I). This suggests that the maturation medium not only improves structural integrity and contraction/relaxation kinetics of the microtissues but also enhances responsiveness to electrical stimulation.

Immunostaining for α-sarcomeric actinin revealed improved sarcomere alignment and Z-band width in microtissues cultured in maturation medium (Figure 5J,K), confirming enhanced structural organization. Electron microscopy further confirmed these findings, revealing a more defined ultrastructure with more abundant myofibrils and a higher abundance of mitochondria interspersed between sarcomeres in the maturation medium group (Figure 5M). 

### 3.7. Improved Physiologically Relevant Response of hiPSC-CMs Cultured in Maturation Medium to Endothelin-1 Hypertrophic Stimulation

**Figure 6:**
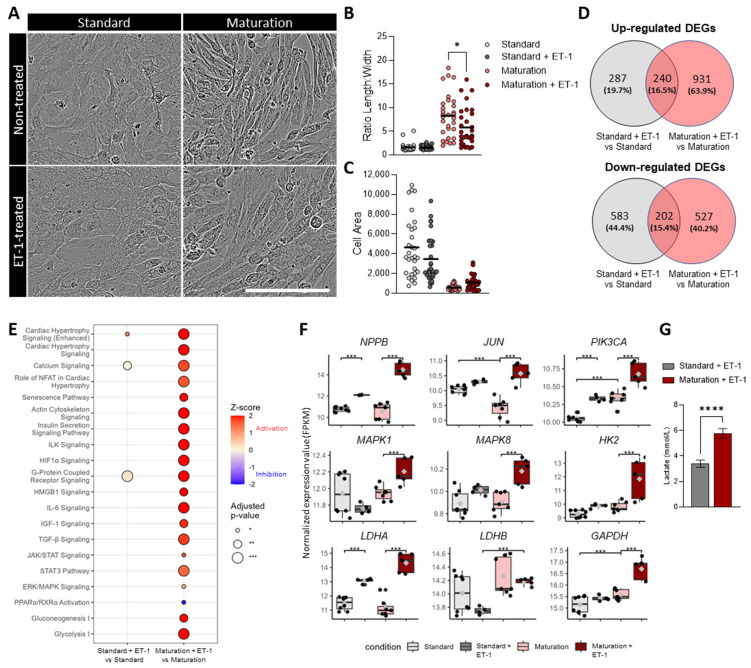
Matured hiPSC-CMs show improved ability to develop hypertrophy features after endothelin-1 (ET-1) stimulation. (**A**) Representative phase contrast images of hiPSC-CMs in standard and maturation medium with and without ET-1 treatment for 48 h. Scale bar: 100 µm. (**B**,**C**) Cell structure characterization in terms of length-to-width ratio (**B**) and cell area (**C**) comparing non-treated and ET-1-treated cells cultured either in standard or maturation medium. (**D**) Venn diagrams of upregulated (**top**) and downregulated (**bottom**) DEGs between ET-1-treated and non-treated hiPSC-CMs in both culture media. (**E**) Significantly activated and inhibited gene expression linked to canonical pathways and disease functions induced by ET-1 treatment in hiPSC-CMs. Terms related to CM hypertrophy, signaling, and metabolism were selected for representation. (**F**) Normalized expression levels of genes associated with cardiac hypertrophy signaling. (**G**) Lactate concentration in the supernatant upon 48 h stimulation with ET-1. Statistical analyses were performed by unpaired Student’s *t*-tests using standard medium as reference or one-way ANOVA with Tukey’s multiple comparisons. Data are represented as mean ± SEM. * *p* < 0.05; ** *p* < 0.01; *** *p* < 0.001; and **** *p* < 0.0001.

Next, we investigated whether the improved maturation features observed in hiPSC-CMs cultured in maturation medium translate into a superior in vitro disease model versus hiPSC-CMs cultured in standard medium. Specifically, we evaluated the pathological response of hiPSC-CMs to 48 h stimulation with ET-1, a potent vasoconstrictor and modulator of cardiac function (Figure 6). ET-1 treatment led to structural alterations in hiPSC-CMs in the maturation medium group, as evidenced by a significant decrease in the length-to-width ratio (Figure 6A,B) and a trend towards increased cell area (Figure 6A,C). Conversely, cells cultured in standard medium exhibited no significant structural changes (Figure 6A–C). RNA sequencing analysis further demonstrated a more pronounced transcriptional effect in cells cultured in maturation medium, with a two-fold increase in the number of DEGs compared to cells cultured in standard medium before ET-1 stimulation (1171 vs. 527 DEGs, Figure 6D). Remarkably, only approximately 15% of the DEGs were common between cells in both media, highlighting distinct responses to ET-1 treatment.

Thorough pathway analysis showed that ET-1 stimulation of hiPSC-CMs cultured in maturation medium significantly activated the cardiac hypertrophy pathway, as well as additional signaling pathways related to hypertrophic response, including calcium, actin cytoskeleton, ILK, HIF1a, G-protein-coupled receptor, HMGB1, and IL-6 signaling, amongst others (Figure 6E). Several metabolic-related pathways, including glycolysis and gluconeogenesis, were also significantly activated. Of the aforementioned pathways, only very few were significantly differentially regulated in hiPSC-CMs cultured in standard medium, confirming that these cells were less responsive to the ET-1 treatment (Figure 6E). In agreement, the expression levels of hypertrophy-related genes including *NPPB*, *JUN*, *PIK3CA*, *MAPK1*, and *MAPK8* were significantly increased upon ET-1 treatment when cells were cultured in maturation medium, reaching higher expression levels than cells cultured in standard medium (Figure 6F). In addition, a significant increase in the expression levels of glycolytic genes (*HK2*, *LDHA*, *LDHB*, *GAPDH*, Figure 6F) and lactate production (Figure 6G) confirms that hiPSC-CMs in maturation medium undergo a more pronounced metabolic switch towards glycolytic metabolism upon ET-1 stimulation in comparison to hiPSC-CMs cultured in standard medium. Overall, these results demonstrate that hiPSC-CMs cultured in maturation medium exhibit a significantly enhanced acute hypertrophic response when stimulated with ET-1, compared to those cultured in maintenance medium, highlighting the superior fidelity and translatability of these cells for in vitro modeling of cardiac disorders.

### 3.8. Enhanced In Vitro-to-In Vivo Correlation Achieved with hiPSC-CM Cultured in Maturation Medium

**Figure 7:**
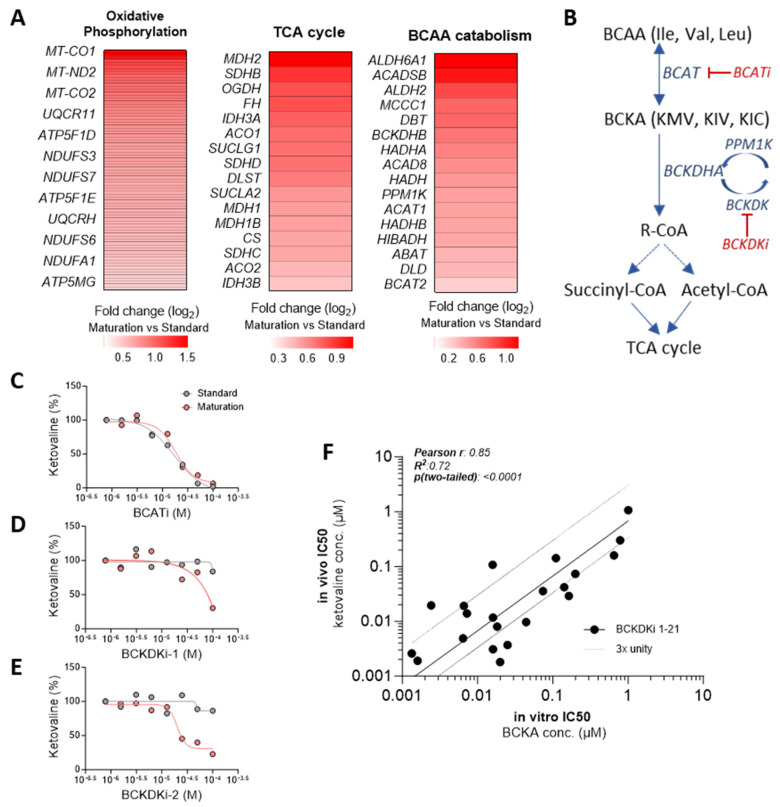
Maturation medium improves correlation between in vitro and in vivo target engagement in metabolic drug screening. (**A**) Heatmap of fold change in expression of genes involved in oxidative phosphorylation, TCA cycle, and BCAA catabolism in cells cultured in maturation medium compared to standard medium, as assessed by RNA-seq. Only genes with FDR < 0.05 are represented. (**B**) Schematic overview of BCAA catabolism pathway highlighting the key enzymes inhibited in the ketovaline assay: branched-chain amino acid transaminases (BCATs) and branched-chain keto acid dehydrogenase kinase (BCKDK). (**C**–**E**) Dose responses on ketovaline concentration as a functional read-out of BCAT inhibition (**C**) and BCKDK inhibition with two inhibitors: BCKDKi-1 (**D**) and BCKDKi-2 (**E**) in standard and maturation medium-treated hiPSC-CMs. (**F**) Correlation between in vitro IC50 of ketovaline concentration in hiPSC-CMs cultured in maturation medium and in vivo IC50 of branched-chain α-keto acid (BCKA) concentration in rat plasma for 21 different BCKDK inhibitors (circles). The dotted lines represent a unity of 3 times in correlation between in vitro and in vivo IC50 values. Pearson correlation shows a significant positive correlation between in vitro and in vivo values.

An additional goal of improving hiPSC-CM maturation is to generate predictive in vitro models for robust drug screening, ultimately reducing the need for animal testing. We have investigated if hiPSC-CMs cultured in maturation medium show enhanced translatability to in vivo drug response when compared to cells cultured in standard medium. We focused on a drug targeting the branched-chain amino acid (BCAA) metabolism, since in addition to upregulation of oxidative phosphorylation- and TCA cycle-related genes, RNA sequencing data unveiled an upregulation of genes associated with the catabolism of BCAAs in hiPSC-CMs cultured in maturation versus standard medium (Figure 7A). 

We started by comparing the response of cells cultured in both media to inhibitors of both branched-chain amino transferase (BCAT), which converts BCAAs into branched-chain keto acids (BCKAs), and branched-chain keto acid dehydrogenase kinase (BCKDK), which regulates the branched-chain α-keto acid dehydrogenase (BCKDH) complex (Figure 7B). Both BCAT and BCKDK inhibitors should decrease the levels of BCKAs, assessed in our assay by the percentage of ketovaline concentration in the medium. Our results demonstrated that BCAT inhibition leads to a dose-responsive reduction in ketovaline concentrations in hiPSC-CMs cultured in both standard and maturation media (Figure 7C). However, a consistent dose-dependent decrease in ketovaline concentrations was exclusively observed upon BCKDK inhibition in hiPSC-CMs cultured in maturation medium (Figure 7D,E). In contrast, hiPSC-CMs cultured in standard medium did not exhibit a significant response to BCKDK inhibition, suggesting an underdeveloped BCAA metabolic pathway in these cells. 

Importantly, we observed a significant positive correlation between the half-maximal inhibitory concentration (IC50) values for ketovaline concentration upon BCKDK inhibition in matured hiPSC-CMs and in vivo IC50 values of BCKAs in rat plasma for 21 different BCKDK inhibitors [41,46,47] (Figure 7F). Such correlation was not possible to obtain using standard medium. This robust in vitro-to-in vivo correlation validates the predictive value of our mature in vitro hiPSC-CM model for drug discovery applications.

## 4. Discussion

A major aim of improving the maturation of hPSC-CMs is to better recapitulate human adult CMs for more translatable target engagement studies, disease modeling, efficacy and safety assessments of potential therapeutic compounds. This study introduces an enhanced and effective maturation strategy of hPSC-CMs by combining ACC2 inhibition with previously reported metabolic and biochemical conditioning. This is to our knowledge the first study to investigate the impact of ACC2 inhibition on hPSC-CM maturation. To simultaneously enhance multiple aspects of CM maturation, we combined pharmacological ACC2 inhibition with established maturation inducers: glucose-depleted medium supplemented with FAs, galactose, and a TID cocktail (T3, IGF-1, and DEX). 

Given that in vivo CM development is accompanied by a metabolic shift towards oxidative metabolism, a prevalent maturation strategy in the literature involves impairing glycolysis by adding FAs with reduced or no glucose, or substituting glucose with galactose [15,17,18,27]. The beneficial effects of thyroid hormones and glucocorticoids on CM functional maturation are also well documented [4,48]. Several studies have shown that a combination of T3, IGF-1, and DEX consistently outperforms single or dual treatments in improving hPSC-CM maturation regarding metabolic, electrophysiological, and contractile properties. Consequently, these components are frequently included in combination in maturation media [5,8].

Despite using this set of well-characterized maturation inducers, our findings reveal that the inclusion of an ACC2i in the medium formulation exerts an additive effect on hiPSC-CM maturation. Specifically, ACC2i supplementation significantly further enhances mitochondrial bioenergetics, shifts reliance towards FAO, increases respiratory reserve capacity, and enhances mitochondrial content and expression levels of oxidative phosphorylation-related genes. Metabolic reserve capacity in particular is a critical indicator of mitochondrial health and efficiency, reflecting the cell’s ability to ramp up energy production in response to increased demand or stress. An enhanced bioenergetic reserve capacity is indicative of metabolic plasticity and is essential for sustaining cellular function under both acute and chronic stress conditions. This capacity plays an important role in mitigating the progression of pathological conditions and is a defining characteristic of adult, metabolically mature, CMs. Furthermore, our results show that ACC2i not only improves metabolic maturation but also enhances calcium transients, accompanied by upregulation of genes linked to calcium handling and cardiac function. While we have not conducted detailed mechanistic studies to determine whether the observed beneficial effects on hiPSC-CM function are directly mediated by ACC2i or are an indirect consequence of enhanced metabolic maturity, the improvements in calcium transients and gene expression are noteworthy. Interestingly, a recent study demonstrated that the functional, metabolic, and transcriptional maturation of hiPSC-CMs are not inherently linked, suggesting that the impact of medium components on distinct maturation features of hiPSC-CMs warrants separate investigation [24]. Future studies focusing on the mechanistic pathways through which ACC2i exerts its effects will be crucial for unraveling the complex interplay between metabolic maturation, transcriptional regulation, and CM function.

We reported a significant increase in the expression of two key transcription factors involved in FA metabolism, *PPARD* and *KLF15*, following ACC2 inhibition. PPARD activation has been shown to upregulate the FAO-linked transcriptional program, increase mitochondrial content, and contribute to hPSC-CM metabolic and contractile maturation [19]. KLF15, primarily investigated for its metabolic effects in the heart, has recently also been implicated as being a key transcription factor driving the maturation of hPSC-CM [49]. Recent studies showed a direct interaction of KLF15 with KCNIP2 (potassium voltage-gated channel-interacting protein 2), which may explain a link between KLF15 and improved ion channel expression and cardiac function in hPSC-CMs. KCNIP2 plays multiple roles in CM function, such as contribution to excitation–contraction coupling through calcium influx modulation, regulation of the fast transient outward K^+^ current, and maintenance of early cardiac repolarization [50]. Knockdown of *KCNIP2* was shown to reduce *SCN5A* (sodium channel protein type 5 subunit alpha, also known as NaV1.5) and *KCND3* (potassium voltage-gated channel subfamily D member 3) expression, leading to decreased calcium transient amplitude [51]. Consistently with an increased expression of *KLF15*, we also observed an upregulation of *KCNIP2*, *SCN5A*, and *KCND3* as well as an enhanced calcium transient amplitude and outward K^+^ current in hiPSC-CMs cultured in maturation versus standard medium. 

In addition to the abovementioned improvements in electrophysiological properties, we also observed that hiPSC-CMs in maturation medium exhibit a more hyperpolarized MDP, faster maximum upstroke velocity, a shorter, more adult ventricular CM-like AP duration, increased expression of genes encoding for K^+^ channels (*KCNQ1*, *KCNJ2*) and the sodium channel *SCN5A*, reduction in pacemaker current channels, and increased I_Na_ and I_K1_ currents, all key characteristics of adult CMs. Kir2.1 (I_K1_) and Nav1.5 (I_Na_) were previously shown to preassemble and travel together to the plasma membrane, which may underlie the here-observed increase in their functional expression [52]. Typically, hiPSC-CMs have a small or even complete lack of I_K1_ current [53]. I_K1_ current in hiPSC-CMs cultured in maturation medium was present at comparable levels as measured in adult human and rabbit ventricular CMs [54]. Remarkably, the I_K1_ current achieved here surpasses the levels reported in hiPSC-CMs subjected to other maturation protocols, such as treatment with T3 and DEX [3] and a metabolic maturation medium containing low glucose, L-lactate, vitamin B12, biotin, creatine monohydrate, taurine, L-carnitine, ascorbic acid, and Albumax [17]. These findings highlight the superior efficacy of our maturation medium formulation containing ACC2i in enhancing the electrophysiological maturity of hiPSC-CMs over more complex medium formulations but also other sophisticated approaches, such as overexpression of I_K1_ channel subunits, proposed to compensate for low I_K1_ current density in hPSC-CMs [55]. In accordance, literature findings suggest that supplements like carnitine, creatine, and taurine, despite their known roles in cellular metabolism, do not significantly impact calcium flux dynamics, impedance, and Nav current recordings in hiPSC-CMs, indicating their dispensability from a cardiac functional perspective [24]. To elucidate the similarities and differences among the compositions of maturation media, we have compared a selection of published media compositions with our own, as summarized in Table S2. This comparison may suggest that addition of ACC2i to other media compositions may offer further benefits for maturation, warranting additional investigation in future research.

Additionally, we observed structural changes in hiPSC-CMs cultured in maturation medium, resulting in a more adult-like CM appearance. These hiPSC-CMs exhibit a more axially elongated shape, greater sarcomere organization and alignment, and increased Cx43 plasma membrane expression. HiPSC-CM in maturation medium also formed superior 3D microtissues, characterized by enhanced contraction/relaxation kinetics and more mature and organized sarcomere structure.

In summary, our study has demonstrated that with the here-presented maturation medium, comprehensive enhancements across a broad set of core aspects of CM maturation are achieved in hiPSC- as well as hESC-derived CMs, including metabolic, functional, electrophysiological, structural, and molecular characteristics. A deeper understanding of the impact of the CM maturation medium, particularly the role of ACC2i, on protein expression and post-translational modifications represents a valuable direction for future research.

Our study further suggests that culture of hiPSC-CMs in maturation medium improves the physiological properties and disease-related readouts in an ET-1-induced hypertrophy assay. ET-1 is known to trigger a hypertrophic response in CMs, characterized by an increase in cell size, protein synthesis, enhanced contractility, reactivation of fetal gene expression, and metabolic shifts including changes in substrate utilization and energy production. These effects are primarily mediated through the activation of mitogen-activated protein kinases (MAPKs) and other signaling pathways. The metabolic adaptations are crucial for meeting the increased energy demand associated with enhanced contractility and hypertrophic growth. In agreement, hiPSC-CMs cultured in maturation medium displayed an augmented response to short-term ET-1 stimulation, indicative of a more mature state. This response included structural changes (e.g., increased cell size), transcriptional alterations (including significant changes in expression of key genes associated with hypertrophy, as well as with signaling pathways related to hypertrophy and metabolic adaptations), and metabolic shifts, suggesting a preference towards glycolytic metabolism. These findings underscore the potential of hiPSC-CMs cultured in maturation medium to generate in vitro cardiac disease models with improved translatability and physiological and pathophysiological characteristics, thereby enhancing the accuracy of drug discovery assessments.

In accordance, we also confirmed that hiPSC-CMs cultured in maturation medium exhibit an improved in vitro-to-in vivo correlation in metabolic responsiveness following drug treatment. Adult CMs are equipped with extensive metabolic machinery, enabling the metabolic processing of diverse substrates for energy production, such as FAs, glucose, and BCAAs. BCAAs, including leucine, isoleucine, and valine, undergo catabolism via a shared pathway in the mitochondria and are subsequently oxidized in the TCA cycle. In the context of pathological cardiac hypertrophy, BCAA catabolism is compromised, leading to reduced expression of catabolic enzymes and accumulation of BCAAs and BCKAs. It has been proposed that enhancing BCAA catabolism through the pharmacological inhibition of BCKDK could mitigate BCAA accumulation, thereby ameliorating heart failure pathophysiology [41]. The strong in vitro-to-in vivo correlation obtained in our studies validates the use of mature hiPSC-CMs as a platform for in vitro screening of BCKDK inhibitors. We suggest that the maturation medium described herein produces hiPSC-CMs with an improved predictive value for drug testing a crucial advancement for translational drug discovery research.

## 5. Conclusions

Our findings collectively confirm the efficacy of the ACC2i-mediated maturation approach in robustly enhancing the maturation of hPSC-CMs. We show that ACC2i supplementation in combination with FAs, galactose, and TID cocktail is sufficient to markedly improve the metabolic, functional, and transcriptional signature of hPSC-CMs, further advancing the application of these cells in cardiac disease modeling and drug discovery. Due to its simplicity, scalability, and robustness, the established medium can be widely applied to support basic research and translational studies using hPSC-CMs cultures. Future research should further explore the application of ACC2i to enhance hPSC-CM maturation in combination with protocols employing other basal culture media and maturation inducers, as well as its impact on a broader range of in vitro cardiac disease models and drug screening platforms.

## Data Availability

The transcriptomics dataset generated for iCell and ChiPS22-derived cardiomyocytes is openly available in FigShare with DOI:10.6084/m9.figshare.25931530, containing raw counts, normalized TPM values, and metadata with a description of the experimental setup.

## Supplementary Materials

The following supporting information can be downloaded at: https://www.mdpi.com/article/10.3390/cells13161339/s1, Figure S1. ACC2 inhibition enhances fatty acid oxidation. Increasing concentrations of ACC2 inhibition is toxic for hiPSC-CMs at concentrations exceeding 5 μM; Figure S2. Enhanced adult-like bioenergetics in hiPSC-CMs cultured in maturation medium; Figure S3. Maturation medium enhances oxidative metabolism, structural organization and expression of cardiac markers in ChiPS22 hiPSC-CMs; Figure S4. Impact of maturation vs standard medium on hiPSC-CMs at the transcriptional level; Figure S5. ACC2 inhibition supplementation improves Ca^2+^ handling properties of hiPSC-CMs; Figure S6. Maturation medium enhances response to hERG blockers on the AP parameters; Figure S7. hiPSC-CMs in maturation medium show more mature ultrastructural properties; Figure S8. Maturation medium improves structural organization, expression of cardiac markers and differentiation towards ventricular phenotype in SA121 hESC-CMs; Table S1. Electrophysiological AP parameters of spontaneously beating hiPSC-CMs cultured in standard and maturation medium; Table S2. Composition of previously published maturation media in comparison to the maturation medium presented in this study. References [5,7,11,15,17,19,21,24,56] are cited in the supplementary materials.
