# Supplementary material for: Enhancing Maturation and Translatability of Human Pluripotent Stem Cell-Derived Cardiomyocytes through a Novel Medium Containing Acetyl-CoA Carboxylase 2 Inhibitor
Source: Cells. 2024 Aug 13;13(16):1339. doi: 10.3390/cells13161339 (PMC11352932; doi:10.3390/cells13161339)
Supplement: Supplementary file 1 [file cells-13-01339-s001.zip › cells-3085458-supplementary.pdf]

Supplementary Materials

Supplementary Tables

Table S1. Electrophysiological AP parameters of spontaneously beating hiPSC-CMs cultured in standard and maturation medium. dV/dt_{max}, AP upstroke velocity; MDP, Maximum diastolic potential (MDP); APA, AP amplitude; DDR, Diastolic depolarization rate; APD20, AP duration at 20% repolarization; APD50, AP duration at 50% repolarization; APD90, AP duration at 90% repolarization. Arrows indicates directionality of the changes in maturation vs standard medium: up/green showing increase and down/red showing decrease. Statistical analyses were performed using unpaired Student's t tests relative to control (standard medium). Data are presented as mean ± SD.

Parameters (Unit)		Standard Medium				Maturation Medium				P value	
		MEAN	±	SD	N	MEAN	±	SD	N		
dV/dt_{max}	($V.s^{-1}$)	23.39	±	25.66	43	68.59	±	43.50	21	↑	<0.0001
Threshold	(mV)	-67.89	±	10.28	43	-76.70	±	4.36	21	↓	0.0002
MDP	(mV)	-74.32	±	8.18	43	-79.17	±	4.00	21	↓	0.0127
APA	(mV)	106.94	±	11.63	43	114.55	±	8.72	21	↑	0.01
DDR	($mV.s^{-1}$)	3.17	±	2.82	43	0.99	±	0.67	21	↓	0.0061
Frequency	(Hz)	0.23	±	0.13	43	0.37	±	0.19	21	↑	<0.0001
APD20	(ms)	239.8	±	92.5	43	93.1	±	30.1	21	↓	<0.0001
APD50	(ms)	527.4	±	144.6	43	182.5	±	54.4	21	↓	<0.0001
APD90	(ms)	828.8	±	176.1	43	357.0	±	69.6	21	↓	<0.0001

Table S2. Composition of previously published maturation media in comparison to the maturation medium presented in this study. [1] Not supplemented (NS) but included in B27, [2] NS but included in basal medium, [3] NS but AlbuMAX contains lipids, [4] NS but included in Chemically defined lipid solution, [5] NS but included in ITS-X (solution containing insulin, transferrin, selenium and ethanolamine). Note: not all media components might be covered in this table, either due to proprietary information related to commercial media, or occasional omissions in literature.

**Component**	(Birket et al., 2015)	(Mills et al., 2017)	(Correia et al., 2017)	(Yang et al., 2019)	(Feyen et al., 2020)	(Murphy et al., 2021)	(Wickramasinghe et al., 2022)	(Fetterman et al., 2024)	**Our medium**
Basal Medium	DMEM (no glucose)	DMEM (no glucose)	RPMI (no glucose)	RPMI	DMEM (no glucose)	RPMI	DMEM	RPMI (no glucose)	RPMI (no glucose)
ACC2i (µM)	0	0	0	0	0	0	0	0	5
PPAR alpha agonist (µM)	0	0	0	0	0	10	0	0	0
PPAR delta agonist (µM)	0	0	0	0	0	0	5	0	0
B27™	0	2x	1x	1x	1x	1x	0	0	1x
BSA (mg/ml)	NS[1]	NS[1]	NS[1]	NS[1]	NS[1]	NS[1]	ND	120	NS[1]
Insulin (µg/ml)	NS[5]	NS[1]	NS[1]	NS[1]	NS[1]	NS[1]	0	10	NS[1]
Glucose (mM)	15	1	0	NS[2]	3	NS[2]	NS[2]	0	0
Galactose (mM)	0	NS[1]	10	NS[1]	NS[1]	NS[1]	NS[1]	2	10
Palmitic acid (µM)	NS[4]	10-100	50	52.5	NS[3]	0	12.5	50	50
Oleic acid (µM)	NS[4]	0	100	40.5	NS[3]	0	12.5	50	100
Linoleic acid	NS[4]	NS[1]	NS[1]	22.5	NS[1,3]	0	12.5	0	NS[1]
Dexamethasone (µM)	1	0	0	0	0	0	0	1	1
IGF1 (ng/ml)	100	0	0	0	0	0	0	0	100
T3 (µM)	0.1	NS[1]	NS[1]	NS[1]	NS[1]	NS[1]	NS[1]	0.1	0.1
L-lactate (mM)	0	0	0	0	10	0	0		0
Vitamin B12 (µM)	0	0	NS[2]	NS[2]	3.69	NS[2]	0	NS[2]	NS[2]
L-carnitine (mM)	0.5	NS[1]	NS[1]	0.12	2	NS[1]	0	2	NS[1]
Ascorbic acid (mM)	0.28	0.2	0	0	0.5	0	0	1.13	0
NEAA	NS[6]	NS[6]	NS[2]	NS[2]	1x	NS[2]	NS[6]	NS[2]	NS[2]
AlbuMAX™ I (%)	0	0	0	0	0.5	0	0	0	0
KnockOut™ Serum (%)	0	0	0	0	1	2	0	0	0
Creatine (mM)	1	0	0	0	0	0	0	5	0
Taurine (mM)	5	0	0	0	0	0	0	5	0
Sodium selenite (ng/ml)	NS[5]	0	0	0	0	0	0	15	0
Transferrin (µg/ml)	NS[5]	NS[1]	NS[1]	NS[1]	NS[1]	NS[1]	NS[1]	20	NS[1]
Pyruvate (mM)	0.5	0	0	0	0	0	0	0	0
Hydroxybutyrate (mM)	0.19	0	0	0	0	0	0	0	0
α-thiolglycerol (µM)	400	0	0	0	0	0	0	0	0
ITS-X	0.1x	0	0	0	0	0	0	0	0
Chemically defined lipids (Life Technologies)	1x	0	0	0	0	0	0	0	0
Trace elements (A,B, and C; Corning)	1x	0	0	0	0	0	0	0	0
Thiazovivin (µM)	0	0	0	0	0	2	0	0	0

Supplementary Figures

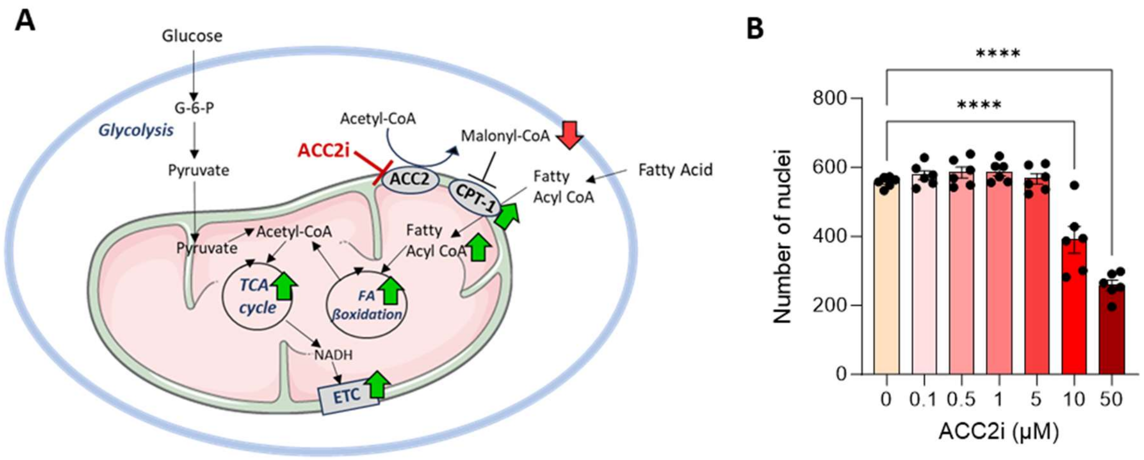

Figure S1. ACC2 inhibition enhances fatty acid oxidation. Increasing concentrations of ACC2 inhibition are toxic for hiPSC-CMs at concentrations exceeding 5uM. A. Schematic showing signaling cascade and mode of action of acetyl-CoA carboxylase (ACC). There are 2 ACC isoforms: ACC1 in cytosol that is dedicated to the rate limiting step of de novo lipogenesis, and ACC2 a mitochondrial membrane associated enzyme that generates malonyl-CoA which in turn allosterically inhibits carnitine palmitoyltransferase (CPT-1), responsible for long-chain fatty-acid transport into mitochondria. Combined ACC1 and 2 inhibition is expected to reduce malonyl-CoA levels and de-novo lipogenesis, and to enhance CPT-1 activity and fatty acid oxidation (FAO). G-6-P (glucose-6-phosphate), TCA cycle (tricarboxylic acid cycle), ETC (electron transport chain). **B.** Impact of increasing doses of ACC2i on hiPSC-CMs viability assessed by number of nuclei after 7 days in culture. Cell toxicity was observed at ACC2i concentrations higher than 5 µm. Source of mitochondria image: Servier Medical Art. Statistical analysis was performed by one-way ANOVA with Tukey's multiple comparisons. Data are presented as mean ± SEM. *$p < 0.05$, **$p < 0.01$, ***$p < 0.001$, and ****$p < 0.0001$.

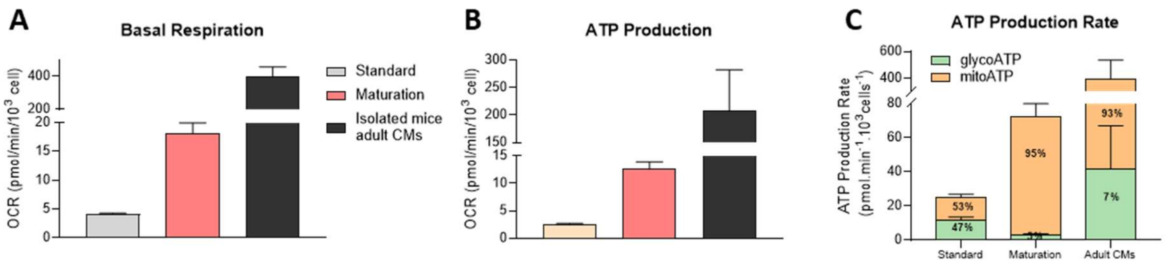

Figure S2. Enhanced adult-like bioenergetics in hiPSC-CMs cultured in maturation medium. Bioenergetics measurements of hiPSC-CMs cultured in standard (grey), maturation medium (red), and freshly isolated adult mouse CMs (black) by Seahorse extracellular flux analyzer. **A.** Basal oxygen consumption rate (OCR); **B.** Proportion of OCR due to ATP production determined with mitochondrial stress test. **C.** Comparison of mitochondrial ATP production and glycolytic ATP production in hiPSC-CMs and adult CMs, showing that hiPSC-CMs in maturation medium show similar mitochondrial and glycolytic ATP production as adult mouse CMs. CMs were isolated from adult mouse hearts using the same method as previously described (Ackers-Johnson et al., 2016). Data were derived from two distinct batches of isolated CMs.

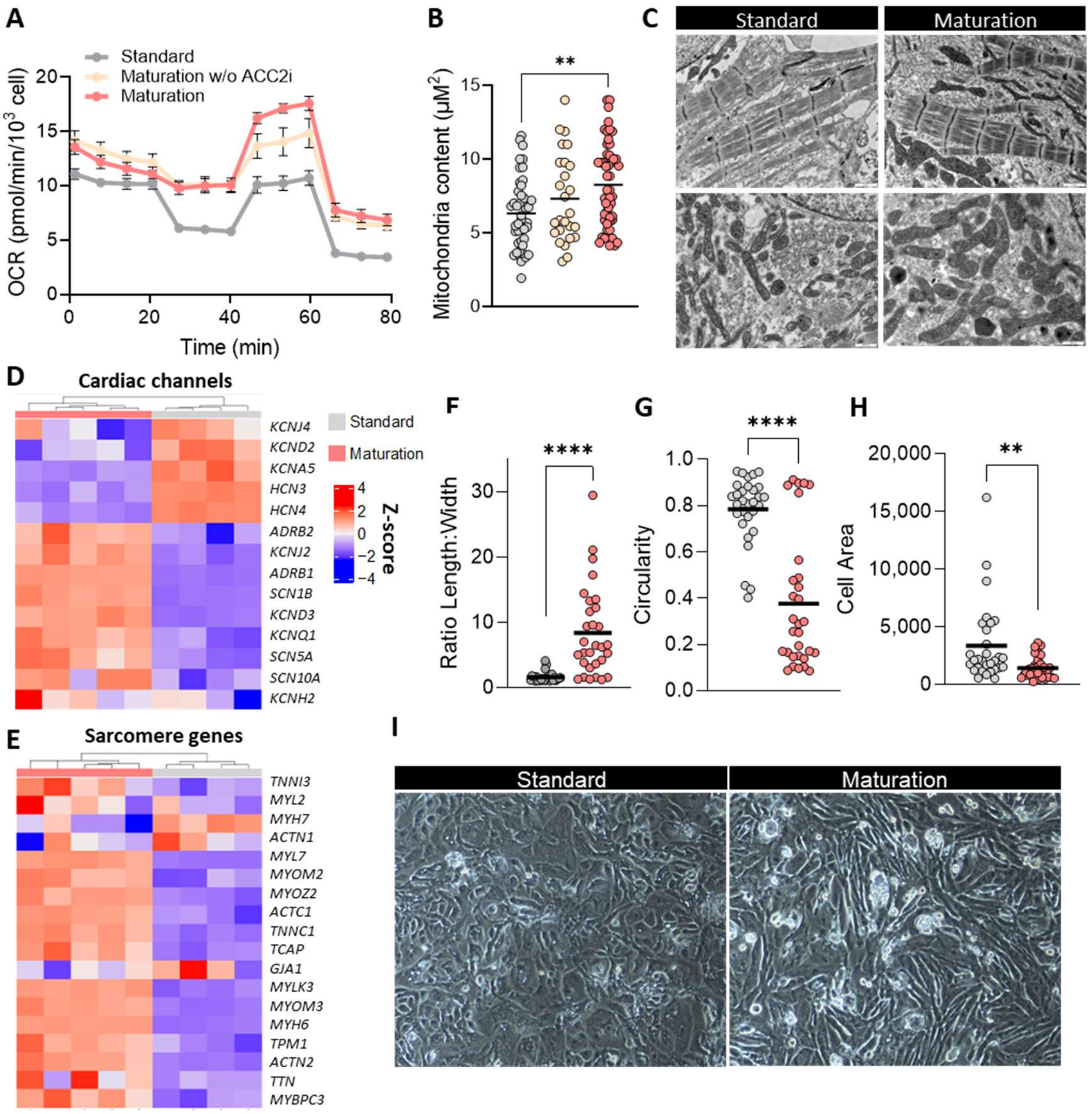

Figure S3. Maturation medium enhances oxidative metabolism, structural organization, and expression of genes related to CM function and structural organization in ChiPS22 hiPSC-CMs. Bioenergetic measurements of ChiPS22 hiPSC-CMs cultured in standard (grey), maturation medium with (red) and without ACC2i (orange) by Seahorse extracellular flux analyzer. **A.** Representative kinetics of the oxygen consumption rate (OCR), showing that cells in maturation medium exhibited more efficient bioenergetics, greater OCR under basal conditions and after mitochondrial uncoupling (n = 3-4 batches). **B.** Quantification of mitochondrial content using total mitochondria area per TEM image. **C.** Representative TEM images showing more organized and aligned sarcomere structures and higher mitochondria content in hiPSC-CMs cultured in maturation medium compared to standard medium. Scale bars: 1 µM (top) and 500 nm (bottom). **D-E.** Heatmap depicting expression levels of cardiac channels and sarcomere genes, respectively. Cell structure characterization in terms of length-to-width ratio **(F)**, circularity index **(G)**, cell area **(H)**. **I.** Representative phase contrast images of cells cultured for 2 weeks in standard and maturation medium. Scale bars: 100 µm. Statistical analyses were performed by unpaired Student's t tests relative to control (standard medium) or one-way ANOVA with Tukey's multiple comparisons. Data are presented as mean ± SEM. $^{*}p < 0.05$, $^{**}p < 0.01$, $^{***}p < 0.001$, and $^{****}p < 0.0001$.

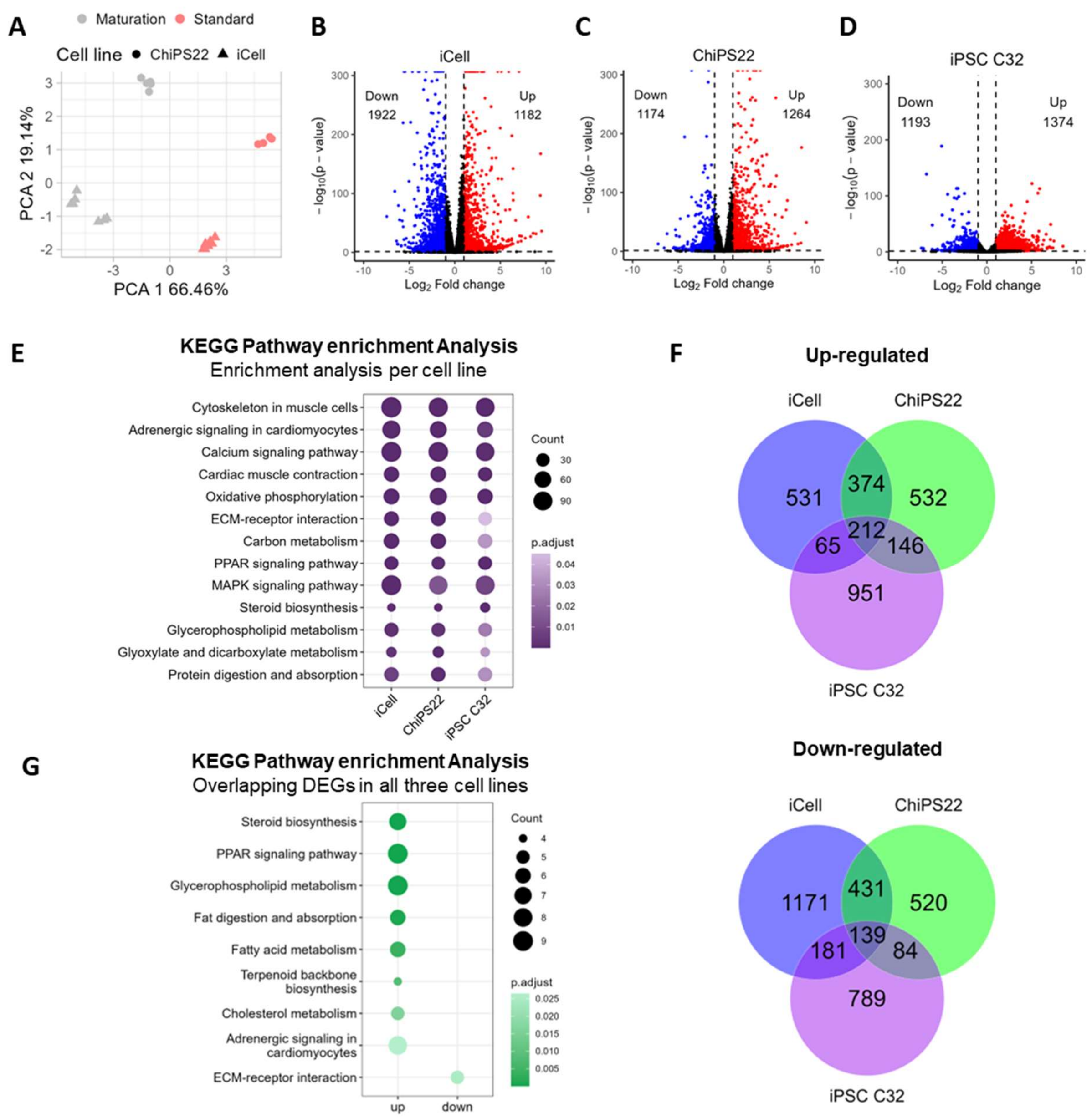

Figure S4. Impact of maturation vs standard medium on hiPSC-CMs at the transcriptional level. A. PCA plot of iCell and ChiPS22 hiPSC-CMs cultured in maturation or standard medium, showing a distinct separation due to culture conditions. **B-D.** Volcano plots showing the number of significantly up- and down-regulated DEGs in CMs derived from three different hiPSC lines and cultured in either maturation or standard medium. **E.** KEGG pathway enrichment analysis of DEGs in hiPSC-CMs cultured in maturation or standard medium. **F.** Venn diagrams showing overlapping up-regulated (top panel) and down-regulated (bottom panel) DEGs for the three hiPSC line derived CMs. **G.** KEGG pathway enrichment analysis of the overlapping DEGs.

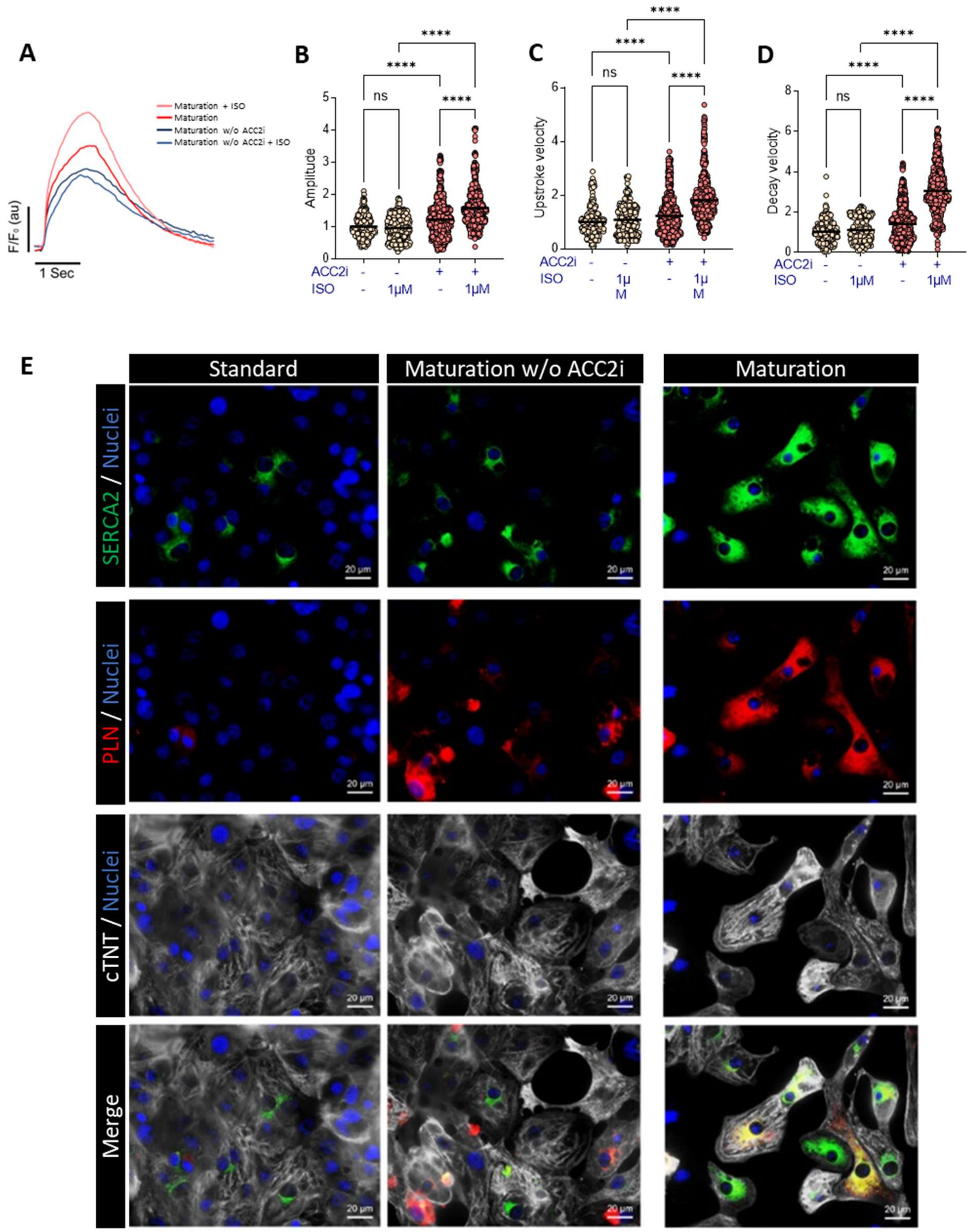

Figure S5. ACC2i supplementation improves Ca^{2+} handling properties of hiPSC-CMs. Calcium imaging analysis showing that ACC2 inhibition (maturation medium) significantly increased amplitude, upstroke, and decay velocity, and improved response to Isoproterenol stimulation in hiPSC-C32 CMs vs maturation medium w/o ACC2i. **A.** Representative Ca^{2+} transients recorded by Fluo-4 AM staining. Ca^{2+} transient amplitude (**B**), upstroke velocity (**C**), and decay velocity (**D**) ofr hiPSC-C32 CMs cultured

in the presence and absence of ACC2i and 1 uM isoproterenol (ISO). HiPSC-C32 CMs cultured in maturation medium without ACC2i and isoproterenol where used as control for data normalization. Each trace is an average normalized F/F0 versus time plot from multiple peaks. Statistical analyses were performed by one-way ANOVA with Tukey's multiple comparisons. Data are presented as mean ± SEM. *p < 0.05, **p < 0.01, ***p < 0.001, and ****p < 0.0001. **E.** Representative fluorescent images of cardiac troponin T (cTnT), sarcoplasmic/endoplasmic reticulum Ca2+-ATPase 2 (SERCA2), and Phospholamban (PLN) in hiPSC-C32 CMs cultured in standard medium, maturation medium without ACC2i, and maturation medium. Scale bars: 20µm.

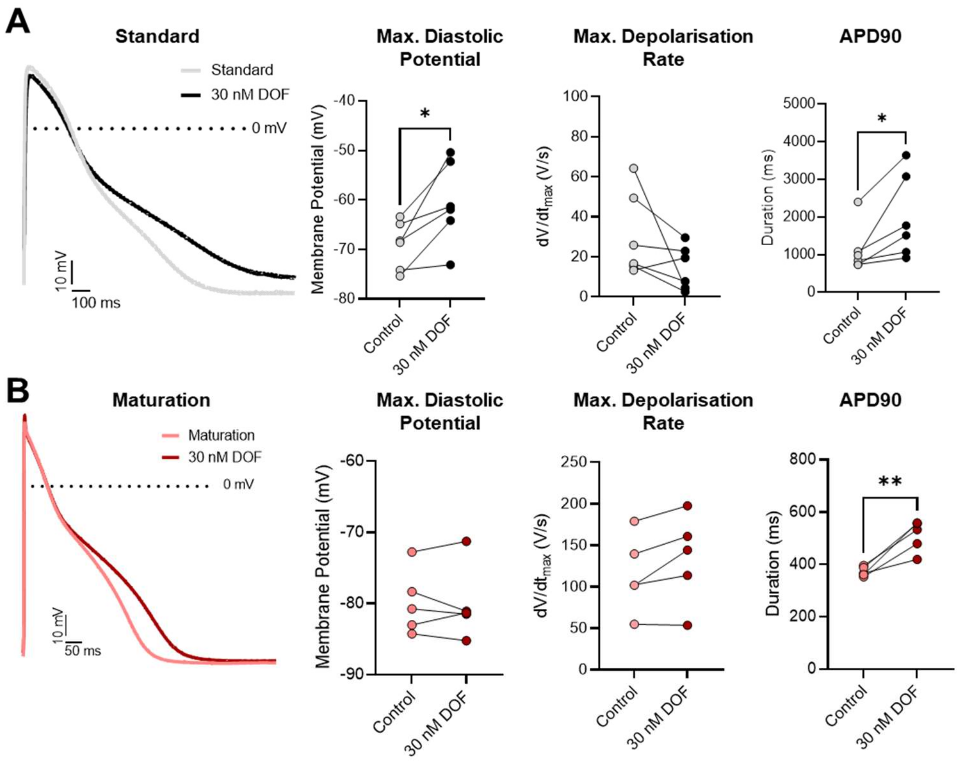

Figure S6. Maturation medium enhances response to hERG blockers. AP recordings were obtained using syncytial manual patch clamp recording to assess the response to dofetilide, a class III antiarrhythmic known to specifically block hERG. Effect of 50 nM dofetilide on maximum diastolic potential (MDP), maximum depolarization rate (MDR), and action potential duration at 90% repolarization (APD90) in standard medium **(A)**, and in maturation medium **(B)**. Statistical analyses were performed using unpaired Student's t tests relative to control (non-treated). ns p>0.05, *p < 0.05, **p < 0.01, and ****p < 0.0001.

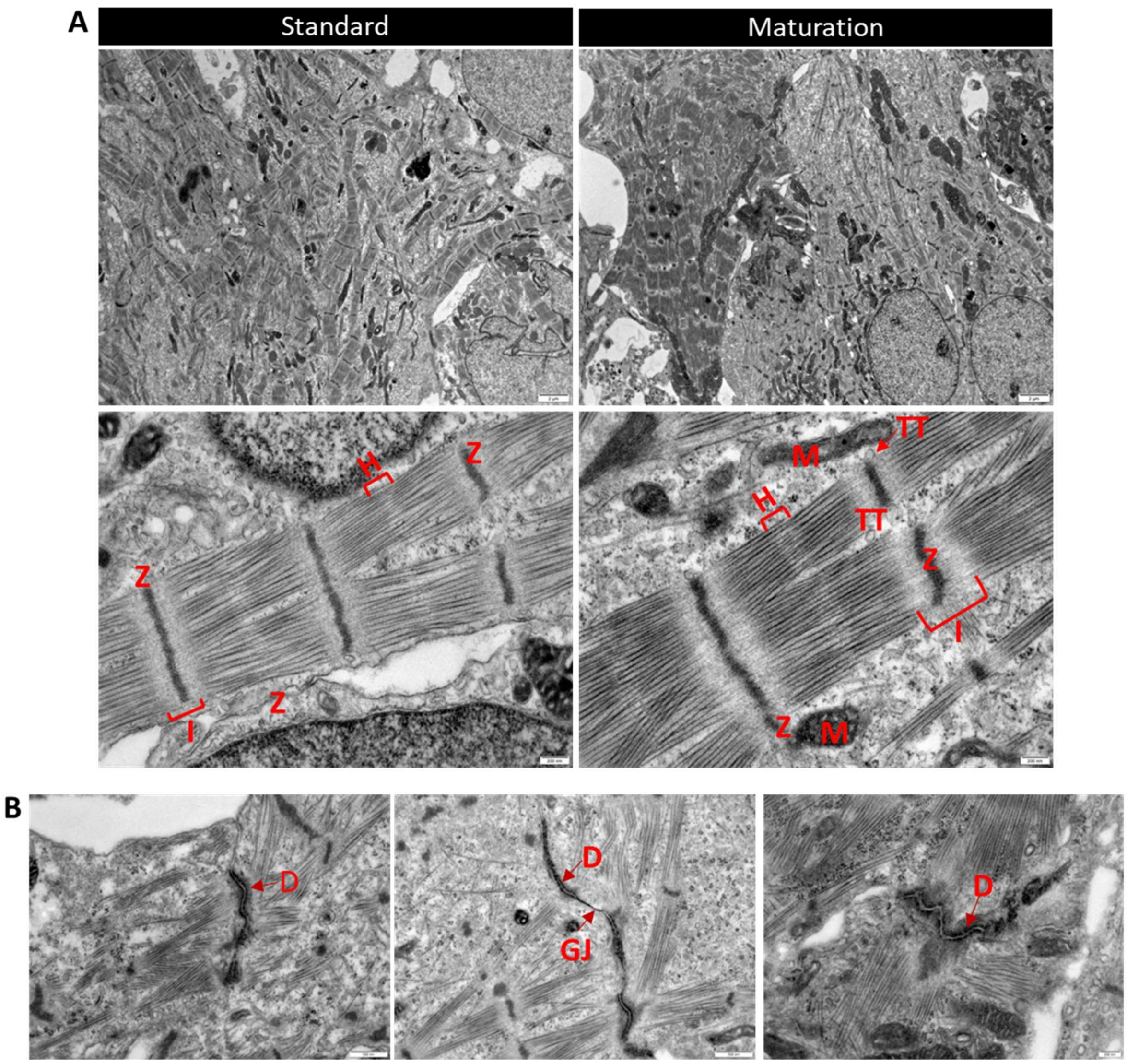

Figure S7. HiPSC-CMs in maturation medium show more mature ultrastructural properties. A. Representative TEM images highlighting myofibril content and structures including Z-discs (Z), sarcomeric bands (I-bands with a H-zone), T-tubules (TT) and mitochondria (M) in iCell-CMs ccultured in either maturation or standard medium. Scale bars: 2 µM (A, top panel) and 200 nm. **B.** Representative TEM images showing a high abundance of desmosomes (D) and Gap/tight junction (GP) -like structures in hiPSC-CMs cultured in maturation medium. Scale bars: 500 nm (left and middle pictures) and 200 nm (right picture).

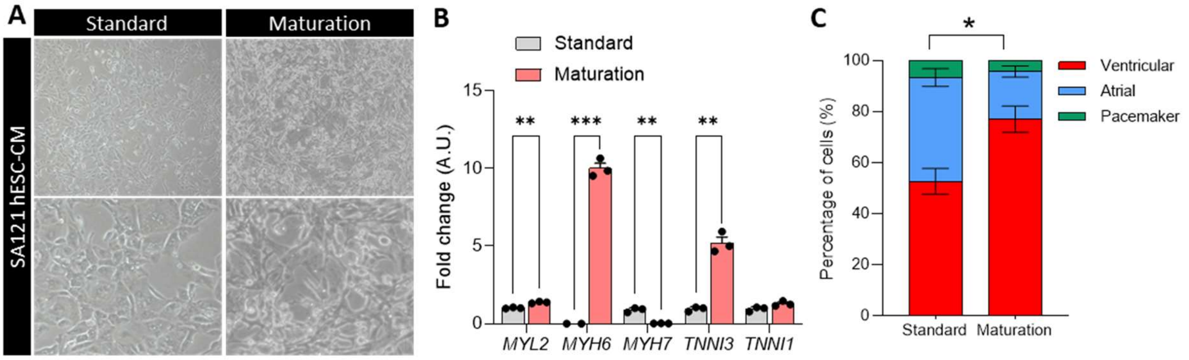

Figure S8. Maturation medium improves structural organization, expression of cardiac markers and differentiation towards ventricular phenotype in SA121 hESC-CMs. A. Bright field images showing SA121 hESC-CMs morphology cultured in standard and maturation media. **B.** Expression of CM genes assessed by qPCR. Gene expression levels were normalized to RPLP0 gene expression. **C.** Single-cell action potential measurements. Percentage of ventricular, atrial, and pacemaker cells when cultured in standard (left) and maturation (right) medium for 2 weeks. A total of 92 hESC-CMs in standard medium vs 90 hESC-CMs in maturation medium were analyzed.

References:

Ackers-Johnson, M., Li, P. Y., Holmes, A. P., O'Brien, S.-M., Pavlovic, D., & Foo, R. S. (2016). A Simplified, Langendorff-Free Method for Concomitant Isolation of Viable Cardiac Myocytes and Nonmyocytes From the Adult Mouse Heart. *Circulation Research*, *119*(8), 909–920. https://doi.org/10.1161/CIRCRESAHA.116.309202

Birket, M. J., Ribeiro, M. C., Kosmidis, G., Ward, D., Leitoguinho, A. R., van de Pol, V., Dambrot, C., Devalla, H. D., Davis, R. P., Mastroberardino, P. G., Atsma, D. E., Passier, R., & Mummery, C. L. (2015). Contractile Defect Caused by Mutation in MYBPC3 Revealed under Conditions Optimized for Human PSC-Cardiomyocyte Function. *Cell Reports*, *13*(4), 733–745. https://doi.org/10.1016/j.celrep.2015.09.025

Correia, C., Koshkin, A., Duarte, P., Hu, D., Teixeira, A., Domian, I., Serra, M., & Alves, P. M. (2017). Distinct carbon sources affect structural and functional maturation of cardiomyocytes derived from human pluripotent stem cells. *Scientific Reports*, *7*(1). https://doi.org/10.1038/s41598-017-08713-4

Fetterman, K. A., Blancard, M., Lyra-Leite, D. M., Vanoye, C. G., Fonoudi, H., Jouni, M., DeKeyser, J. M. L., Lenny, B., Sapkota, Y., George, A. L., & Burridge, P. W. (2024). Independent compartmentalization of functional, metabolic, and transcriptional maturation of hiPSC-derived cardiomyocytes. *Cell Reports*, *43*(5). https://doi.org/10.1016/j.celrep.2024.114160

Feyen, D. A. M., McKeithan, W. L., Bruyneel, A. A. N., Spiering, S., Hörmann, L., Ulmer, B., Zhang, H., Briganti, F., Schweizer, M., Hegyi, B., Liao, Z., Pölönen, R. P., Ginsburg, K. S., Lam, C. K., Serrano, R., Wahlquist, C., Kreymerman, A., Vu, M., Amatya, P. L., … Mercola, M. (2020). Metabolic Maturation Media Improve Physiological Function of Human iPSC-Derived Cardiomyocytes. *Cell Reports*, *32*(3). https://doi.org/10.1016/j.celrep.2020.107925

Mills, R. J., Titmarsh, D. M., Koenig, X., Parker, B. L., Ryall, J. G., Quaife-Ryan, G. A., Voges, H. K., Hodson, M. P., Ferguson, C., Drowley, L., Plowright, A. T., Needham, E. J., Wang, Q. D., Gregorevic, P., Xin, M., Thomas, W. G., Parton, R. G., Nielsen, L. K., Launikonis, B. S., … Hudson, J. E. (2017). Functional screening in human cardiac organoids reveals a metabolic mechanism for cardiomyocyte cell cycle arrest. *Proceedings of the National Academy of Sciences of the United States of America*, *114*(40), E8372–E8381. https://doi.org/10.1073/pnas.1707316114

Murphy, S. A., Miyamoto, M., Kervadec, A., Kannan, S., Tampakakis, E., Kambhampati, S., Lin, B. L., Paek, S., Andersen, P., Lee, D. I., Zhu, R., An, S. S., Kass, D. A., Uosaki, H., Colas, A. R., & Kwon, C. (2021). PGC1/PPAR drive cardiomyocyte maturation at single cell level via YAP1 and SF3B2. *Nature Communications*, *12*(1). https://doi.org/10.1038/s41467-021-21957-z

Wickramasinghe, N. M., Sachs, D., Shewale, B., Gonzalez, D. M., Dhanan-Krishnan, P., Torre, D., LaMarca, E., Raimo, S., Dariolli, R., Serasinghe, M. N., Mayourian, J., Sebra, R., Beaumont, K., Iyengar, S., French, D. L., Hansen, A., Eschenhagen, T., Chipuk, J. E., Sobie, E. A., … Dubois, N. C. (2022). PPARdelta activation induces metabolic and contractile maturation of human pluripotent stem cell-derived cardiomyocytes. *Cell Stem Cell*, *29*(4), 559-576.e7. https://doi.org/10.1016/j.stem.2022.02.011

Yang, X., Rodriguez, M. L., Leonard, A., Sun, L., Fischer, K. A., Wang, Y., Ritterhoff, J., Zhao, L., Kolwicz, S. C., Pabon, L., Reinecke, H., Sniadecki, N. J., Tian, R., Ruohola-Baker, H., Xu, H., & Murry, C. E. (2019). Fatty Acids Enhance the Maturation of Cardiomyocytes Derived from Human Pluripotent Stem Cells. *Stem Cell Reports*, *13*(4), 657–668. https://doi.org/10.1016/j.stemcr.2019.08.013
